# Large-scale plant genomic identification and analysis uncover *ASMT/COMT* copy number variation driving melatonin dosage balance

**DOI:** 10.1093/hr/uhaf348

**Published:** 2025-12-18

**Authors:** Shuotong Liu, Pei Yu

**Affiliations:** SDU-ANU Joint Science College, Shandong University, Weihai 264209, China; SDU-ANU Joint Science College, Shandong University, Weihai 264209, China

## Abstract

ASMT/COMT, as a key rate-limiting enzyme regulating melatonin biosynthesis, has garnered significant attention. This study investigates the evolutionary mechanisms of the *ASMT/COMT* gene family in melatonin biosynthesis. A total of 28 010 *ASMT/COMT* genes from 1052 species were identified through an integrated approach combining large-scale identifications and analyses. At the pan-genome level, we identified 5186, 336, 2137, and 1814 *ASMT/COMT* genes respectively in *Triticum aestivum*, *Aegilops tauschii*, diploid and tetraploid *Solanum tuberosum* haplotype genomes (247, 86, 670, and 96 orthologous gene groups). Expansion patterns of the *ASMT/COMT* gene family were explored through synteny networks in 104 Poaceae and 88 Solanaceae plants. Further investigation of copy number variation (CNV) in the 1052 species, along with a focused analysis of hexaploid wheat and its diploid progenitor *Ae. tauschii*, indicated a functional divergence linked to gene dosage. The catalytically efficient COMT is maintained at low-copy conditions, whereas the less active ASMT is amplified under high-copy conditions. Intriguingly, in polyploid potatoes, the total *ASMT/COMT* copy number was lower in tetraploids than in diploids, suggesting a distinct dosage balance mechanism operating in polyploids. In contrast, the melatonin receptor *CAND2* consistently remained in a low-copy state, with no significant correlation to *ASMT/COMT* copy number. Expression analysis revealed that *COMT* is generally expressed at higher levels than *ASMT*, highlighting a compensatory relationship between gene dosage and transcriptional regulation. Collectively, our findings uncover a dosage balance mechanism that fine-tunes melatonin biosynthetic homeostasis through coordinated CNV and expression regulation, offering a new perspective on the evolution of metabolic enzymes.

## Introduction

The dosage balance hypothesis provides a key framework for understanding gene retention and expansion after whole-genome duplication (WGD) events, offering critical insights into processes in plant adaptation and stress resistance [1–5]. The complexity of the dosage balance hypothesis is manifested across multiple levels: gene dosage effects are not only governed by the coordinated expression of protein complex subunits but also shaped by nonlinear characteristics and functional divergence of paralogous genes, collectively defining gene retention patterns [6, 7]. While this model has been extensively applied to diverse systems, including protein complex subunits and miRNA regulatory networks, some gaps persist in its theoretical framework [6, 8–10].

Notably, plant hormones, as quintessential dosage-sensitive regulators of growth and development, exhibit dynamic interdependencies between dosage variations in their biosynthetic enzymes and hormonal homeostasis [11]. This interplay offers a novel avenue for investigating the evolutionary trajectories of plant enzyme gene families and expanding the theoretical boundaries of the dosage balance hypothesis. Focusing on melatonin, a vital plant hormone, and its rate-limiting biosynthetic enzymes, the N-acetylserotonin O-methyltransferase (ASMT)/caffeic acid O-methyltransferases (COMT) gene family, this study aims to establish regulatory patterns linking gene dosage, enzyme activity, and hormone homeostasis. By unraveling how gene duplication events modulate hormonal metabolic networks, we seek to refine the applicability of the dosage balance hypothesis in the context of secondary metabolic regulation.

Melatonin, the core hormonal molecule under investigation, is an antioxidant that controls reactive oxygen species (ROS) and reactive nitrogen species (RNS) as well as other free radicals and oxidation molecules in plant cells [12, 13]. Studies have indicated that applying exogenous melatonin can boost the activity of key antioxidant enzymes, such as superoxide dismutase (SOD) and catalase (CAT), thereby strengthening plant resilience under various environmental stresses [14, 15]. The identification of downstream signaling chain elements of melatonin, *Cullin Associated and Neddylation Dissociated 2* (*CAND2*) in *Arabidopsis thaliana*, gives a key piece of evidence that melatonin is a plant hormone [15, 16]. As a newly recognized phytohormone, melatonin actively regulates fundamental physiological processes, including root morphogenesis, seed viability, and stomatal gas exchange, (H_2_O/CO_2_), through conserved signaling pathways [15, 17].

Melatonin biosynthesis involves four enzymatic reactions catalyzed by at least seven enzymes, including tryptophan decarboxylase (TDC), tryptophan hydroxylase (TPH), tryptamine 5-hydroxylase (T5H), serotonin N-acetyltransferase (SNAT), ASMT, and COMT [13, 18, 19]. Among them, ASMT/COMT serves as the rate-limiting enzyme and has drawn considerable attention in recent studies across various plant species [20–26]. In *Poncirus trifoliata*, the *PtABF4-PtbHLH28-PtCOMT5* molecular module plays a vital role in promoting drought tolerance and root development in regulating melatonin accumulation [27]. In *Solanum lycopersicum*, NO accumulation triggered by saline–alkali stress stimulates COMT transcription and melatonin biosynthesis, which in turn alleviates protein nitrosation damage by scavenging excess NO [28]. In *Triticum aestivum* (bread wheat), *TaASMT3* significantly enhanced resistance to stripe rust by promoting melatonin biosynthesis [29]. Furthermore, dynamic metabolite QTL analyses have identified *ASMT/COMT* as key genes involved in melatonin biosynthesis [30].

Studies have shown that the expansion of the *ASMT/COMT* gene family is widespread across multiple species, despite variations among species [18, 20, 22, 25, 31]. Basic identifications and analyses of the *ASMT/COMT* gene family are already underway in soybean (*Glycine max*) [32], tea (*Camellia sinensis*) [25], rice (*Oryza sativa*) [21], walnut (*Juglans regia*) [31], and comparative analyses across *Arabidopsis thaliana*, tomato, rice, and sorghum (*Sorghum bicolor*) [33]. The expansion of this gene family is mainly driven by mechanisms such as fragment replication and tandem duplication, and the *K*a/*K*s value is generally <1, indicating that it is mainly subject to purification selection during evolution to maintain the conservation of gene function. Evolutionary tracing studies have revealed that *ASMT* in plants originated from horizontal gene transfer (HGT) during early endosymbiotic events [26]. Following their acquisition, functional divergence of *ASMT* genes was driven by WGD events, contributing to the adaptive evolution of land plants [26]. In previous studies, the authors supported that *COMT* evolved from *ASMT* through WGD based on the exclusion of retroposition and local duplication mechanisms, evidence for the prevalence of WGD in plant evolution, and the evolutionary pattern of *COMT* retaining the original function of *ASMT* while acquiring new lignin biosynthesis capabilities [26].

Although the traceability analysis of *ASMT/COMT* has made great progress, there are still many deficiencies. Some studies on ASMT and COMT enzyme systems have treated them as independent gene families for functional elucidation, but few have integrated and compared them [18, 20, 22, 25, 31]. This separation state of research methods hinders the in-depth interpretation of their functional synergy and evolutionary link. This study is the joint analysis of ASMT and COMT, and the key idea is whether there is an association between overall copy numbers for these two genes and which of the two enzymes is then favored.

Unlike the experimental verification, a fast and cost-effective strategy is to carry out large-scale gene identification at the genome-wide level, reconstruct the phylogenetic framework, and then reveal the patterns of conservation and variation through cross-species comparative analysis, thereby systematically analyzing the functional divergence and evolutionary trajectory of gene families. The breakthroughs in genomics technology in recent years have provided a brand-new perspective for dissecting the above-mentioned evolutionary mechanisms [34]. Currently, >1000 plant species have had their genomes assembled at the chromosome level, covering key evolutionary nodes from mosses to angiosperms [34]. More notably, the pan-genome analysis of multiple species, including wheat, potato (*S. tuberosum*), and the model plant *A. thaliana*, has been completed, providing a revolutionary tool to break through the genetic blind spots caused by a single-reference genome [35–43]. These multiomics data not only constructed a panoramic map spanning 450 million years of plant evolution but also, through cross-species comparison of synteny blocks, revealed the phased contributions of ancient polyploidization events to the expansion of metabolic gene families.

In this study, we identified 28 010 *ASMT/COMT* gene family members across 1052 plant genomes through genome-wide scanning and analysis. Additionally, 2240 *CAND2* gene family members, which encode the melatonin receptor, were identified for copy number variation (CNV) comparison. Based on these findings, we proposed the hypothesis that CNV of *ASMT/COMT* genes drives melatonin dosage balance. The collinear networks of 88 Solanaceae plants (1608 nodes or genes and 34 890 edges or gene pairs) and 104 Poaceae plants (2759 nodes or genes and 32 194 edges or gene pairs) provide insights into the expansion mechanism of the *ASMT/COMT* gene family, becoming a bridge connecting the pan-genome and large-scale identification analysis. With the pan-genome serving as a new reference, datasets from 36 wheat varieties, 7 *Ae. tauschii* accessions, 48 diploid potatoes, and 11 tetraploid potatoes were employed to further test the hypothesis that ploidy-induced CNV contributes to variation in the *ASMT/COMT* gene family in wheat and potato. Finally, transcriptome datasets of *ASMT/COMT* from *Arabidopsis*, wheat, and tomato were employed in the analysis to ensure the robustness of evidence chains. This study provides new insights into the evolutionary dynamics and functional mechanisms of the *ASMT/COMT* gene family and offers fresh perspectives into the holistic framework of gene dosage balance patterns and their evolutionary implications.

## Results

### Large-scale identification and analysis uncovering the patterns of *ASMT/COMT* copy number variation

**Figure 1:**
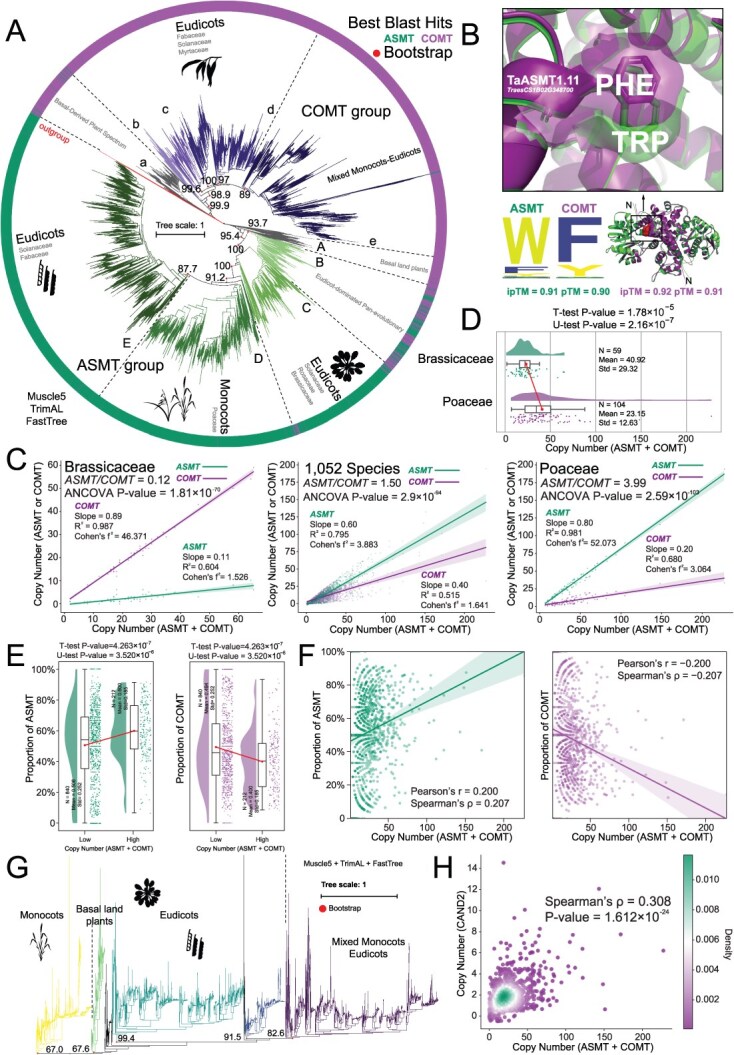
*ASMT/COMT* and *CAND2* phylogenetic and CNV analysis from large-scale cross-species perspective. (A) Phylogenetic tree of the *ASMT/COMT* gene family members across 1052 plant species. The rooted maximum-likelihood (ML) tree was constructed from the full length of 28 010 ASMT/COMT proteins from 1052 species using the ‘LG’ model in FastTree. The specific software used is marked in the figure. **(B)** Identification of key differentiation sites between ASMT and COMT followed by mutation prediction and structural analysis. **(C)** Relationship between the total number of *ASMT/COMT* gene family and the copy number of subfamily. **(D)** Comparison of the *ASMT/COMT* copy number between Poaceae and Brassicaceae family species. Poaceae family is one of high-copies representers and the other is one of low. **(E)** Comparison of high- or low-copy species groups with ASMT/COMT CNV patterns. A total of 1052 species were divided into high- (≥38) and low- (≤37) copy groups based on *K*-means, and the differences of *ASMT/COMT* CNV patterns (the proportion of *ASMT* or *COMT*) between groups were compared. **(F)** Linear fit of the *ASMT/COMT* CNV pattern in 1052 species. The *x*-axis represents total copy number of *ASMT/COMT* and the *y*-axis represents the proportion of *ASMT* or *COMT*. **(G)** Phylogenetic tree of melatonin receptor *CAND2* proteins in 1052 species. The construction method is consistent with Fig. 1A. **(H)** Spearman correlation analysis of copy numbers between *CAND2* and *ASMT/COMT* gene families across 1052 species.

A total of 28 010 *ASMT/COMT* genes from 1052 species were identified through an integrated approach combining large-scale identifications and analyses. Phylogenetic analysis revealed that ASMT and COMT proteins formed two distinct clades and exhibited a well-defined evolutionary gradient from basal outgroups to crown groups (Fig. 1A, Tables S1 and S2). The basal groups at the bottom included algae, ANA-grade basal angiosperms, and gymnosperms, while the upper portion was dominated by major clades of higher plants such as monocots and eudicots (Fig. 1A). Notably, monocots and eudicots formed distinct clusters, revealing significant evolutionary divergence in *ASMT/COMT* genes between these two major plant lineages and suggesting that they have undergone markedly different evolutionary trajectories. Further analysis showed that the *COMT* subfamily exhibited relatively higher evolutionary conservation compared to *ASMT*, primarily reflected in the ‘e’ subgroup, which contained gene members from both monocots and eudicots and demonstrated a phylogenetically intermixed pattern (Fig. 1A).

Based on the massive sequence data, machine learning algorithms such as random forest [44] and XGBoost [45] were used to identify the key loci driving the evolutionary divergence of ASMT/COMT (Fig. 1B, Table S3). To ensure data reliability, the *ASMT/COMT* gene family members whose phylogenetic status was consistent with sequence similarity were selected as the training dataset. This approach led to the identification of 13 candidate conserved amino acid residues (Table S3). Manual functional evaluation revealed a key residue, tryptophan (W) in ASMT or phenylalanine (F) in COMT at the 200th position’s mutation (after alignment trimming). Subsequently, mutation simulations at the key sites of the representative ASMT structure suggest that the mutation may create an enlarged substrate-binding cavity and a more stable hydrophobic environment for the substrate, owing to the increased hydrophobicity and the reduced side chain volume of phenylalanine compared to tryptophan (Fig. 1B and Table S3). And the model was basically accurate before (ipTM = 0.91, pTM = 0.90) and after (ipTM = 0.92, pTM = 0.91) the mutation (Fig. 1B).

To further investigate the CNV characteristics of *ASMT/COMT* genes, we systematically analyzed the copy numbers of both gene types and constructed linear regression models based on total copy numbers (Fig. 1C and D). During the process of increasing the total copy number of *ASMT/COMT*, the growth rate of the *ASMT* subfamily is much higher than that of the *COMT* subfamily (in 1052 species, ASMT’s slope = 0.60; COMT’s slope = 0.40; ASMT’s slope/COMT’s slope = 1.50; *P* = 2.9 × 10^−94^) (Fig. 1C). High-copy species are similar (in Poaceae species, ASMT’s slope = 0.80; COMT’s slope = 0.20; ASMT’s slope/COMT’s slope = 3.99; P-value = 2.59 × 10−103); however, in the lower copied families, such as the Brassicaceae, the phenomenon is the opposite of the above (ASMT’s slope = 0.11; COMT’s slope = 0.89; ASMT’s slope/COMT’s slope = 0.12; *P*-value = 1.81 × 10^−70^) (Fig. 1C and D). And the CNVs of the *ASMT/COMT* gene family in 1052 species were analyzed based on *K*-means clustering algorithm (*k* = 2), and they were divided into a high-copy group (*n* = 212) and a low-copy group (*n* = 840) (Fig. 1E). Statistical analyses, including independent sample *t*-tests (*P-*value = 4.263 × 10^−7^) and Mann–Whitney *U* tests (*P-*value = 3.520 × 10^−6^), confirmed that the relative abundance of the *ASMT/COMT* gene family members differed significantly between the two groups (Fig. 1E and F). Correlation analysis further revealed a positive association between the ratio of *ASMT* or *COMT* genes and the total copy number of the gene family (Pearson *r* = 0.200, *P-*value = 6.48 × 10^−11^; Spearman ρ = 0.207, *P-*value = 1.09 × 10^−11^) (Fig. 1E and F).

To further validate the evolutionary relationship between *ASMT/COMT* genes’ copy number and melatonin metabolism, we conducted comparative analyses across 1052 plant species possessing the melatonin receptor gene *CAND2* (Fig. 1G, Tables S4 and S5). In total, 2240 *CAND2* gene family members were identified from these species. Spearman’s rank correlation analysis revealed a statistically significant but relatively weak positive correlation between the CNV of *ASMT/COMT* and *CAND2* (ρ = 0.308, *P*-value = 1.612 × 10^−24^) (Fig. 1H). At the species level, melatonin receptor copy numbers showed remarkable conservation, with most species maintaining around two copies and the maximum not exceeding 20 (Fig. 1H). These findings suggest that while *ASMT/COMT* genes exhibit coordinated expansion with melatonin receptor genes, this coamplification effect remains limited in magnitude across the majority of species.

Given the copy number comparison between Brassicaceae and Poaceae plants in Fig. 1C and D, we took advantage of the high-quality sequencing and complete pan-genomes of *A. thaliana* to systematically analyze CNV patterns of the *ASMT/COMT* gene family across 59 Brassicaceae plants and the Arabidopsis pan-genome. Cross-species collinear networks and phylogenetic relationships of *ASMT/COMT* in 59 Brassicaceae plants showed a collinearity relationship between *ASMT* and *COMT*, which is in line with the fact that *ASMT* is the ancestral gene (Fig. S1A). Furthermore, we observed that *ASMT* (*AT4G35160*) is collinear with *AT1G21100* (*IGMT1*) [46], a paralog of *COMT* (*AT5G54160*) (Fig. S1B). Whole-genome identification incorporating pan-genomic data revealed that the overall CNV pattern of *Arabidopsis AtASMT/AtCOMT* genes remained largely unchanged compared to that derived from the reference genome alone, consistent with findings from the core genome analysis (Fig. S1C–S1F and Table S6).

### Collinearity analysis revealing the expansion of *ASMT/COMT* in Poaceae

To deeply investigate the evolutionary divergence of relevant gene families between monocots and dicots, we focused on representative taxa: the Poaceae as the model for monocots, with wheat (*T. aestivum*) as the primary representative species, and the Solanaceae as the model for dicots, with potato (*S. tuberosum*) as the primary representative species, for comparative analysis. This strategy of focusing on representative lineages allowed for a clearer elucidation of key patterns.

**Figure 2:**
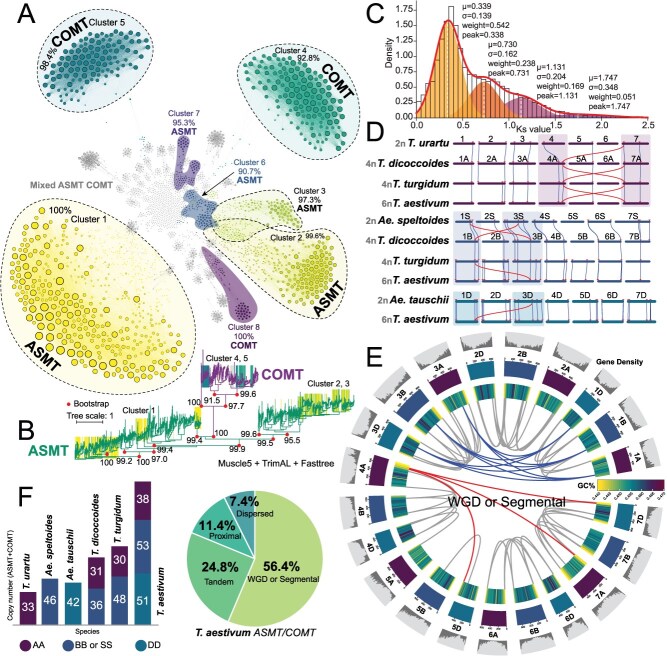
Evolutionary transitions of the *ASMT/COMT* gene family: Collinearity analysis in Poaceae. **(A)** Cross-species collinear networks of *ASMT/COMT* in 104 Poaceae species. **(B)** Unrooted phylogenetic tree of ASMT/COMT proteins in 104 Poaceae species. **(C)** Patterns of the *ASMT/COMT* gene family differentiation among gramineous species were judged based on Ks peak values. The solid line represents the overall GMM fit curve, four peaks indicate individual Gaussian components, and vertical dashed lines mark the peak positions of each component and the global peak. The optimal number of components was automatically selected using the BIC criterion. **(D)** Subchromosomal synteny conservation tracing *ASMT/COMT* evolution from ancestral to modern wheat. **(E)** Collinearity analysis of *TaASMT/TaCOMT* in wheat revealed that WGD events drive *ASMT/COMT* paralog expansion. IWGSC RefSeq Annotation v1.1 was used to perform analyses. **(F)** Mechanistic hierarchy of *ASMT/COMT* expansions: high conservation of subgene-biased WGD paralogs from ancestral to modern wheat. AA, BB or SS, and DD respectively represent the subchromosomal categories of wheat or the ancestral species of wheat. The bar chart shows the copy numbers of *ASMT/COMT* in these species, where different subchromosomes are distinguished using patterned fills.

The collinearity network analysis not only confirmed the homologous relationship between *ASMT* and *COMT* genes but also revealed their significant evolutionary divergence (Fig. 2A and B and Table S7). Following gene duplication and expansion events, *ASMT* and *COMT* genes were distinctly classified into mainly eight characteristic subgroups (Fig. 2A), with Cluster 1 (100% *ASMT*), Cluster 2 (99.6% *ASMT*), and Cluster 3 (97.3% *ASMT*) dominated by *ASMT*, while Cluster 4 was predominantly *COMT* (92.8%). Combined with the phylogenetic tree, the cluster corresponding to ASMT and COMT also shows significant differentiation (Fig. 2B). As an *ASMT*-dominant family, the synteny network subgroups formed by *ASMT* expansion in Poaceae were larger in scale than those of *COMT*. Notably, although *ASMT* and *COMT* genes exhibited highly specialized distribution patterns across the four subgroups, collinearity network analysis still detected weak homologous relationships between them (Fig. 2A). These faint but persistent collinearity signals suggest that despite significant divergence following gene duplication and expansion events, the two gene families retain evidence of their shared evolutionary origin. We identified the prominent synonymous substitution rate (*K*s) peaks at 0.338 (μ = 0.339, σ = 0.139, weight = 0.542), 0.731 (μ = 0.730, σ = 0.162, weight = 0.238), and 1.31 (μ = 1.131, σ = 0.204, weight = 0.169) for this gene family in grasses by comparing *K*s distributions of orthologous genes across species (Fig. 2B and Table S8). Based on the two estimated average annual nucleotide mutation rates of Poaceae, the main *K*s peak corresponds to a divergence time of ~26 million years ago (Mya).

To investigate the evolutionary dynamics of the *ASMT/COMT* gene family under polyploidization, we constructed a subgenome-scale collinearity network and performed intragenomic homology comparisons. At the subgenomic level, the *ASMT/COMT* gene family shows highly conserved evolutionary features (Fig. 2C and Table S9). Collinearity analysis showed that the family genes were generally retained during polyploidy, and local expansions were detected in chromosomes 2 and 5 of the B genome (Fig. 2D). Comparison of collinearity with *T. turgidum* revealed additional collinearity in hexaploid wheat, suggesting that this expansion may have occurred during the evolutionary stage from tetraploid to hexaploid wheat. Notably, subgenome D showed the highest gene retention level (Fig. 2D). This higher retention could be because the D subgenome is the most recently acquired, leaving less time for gene fractionation compared to the A and B subgenomes.

Cross-genome collinearity analysis showed that *ASMT/COMT* genes were highly conserved on chromosomes 1 and 3, which were stable within the wheat genome and across species (Fig. 2D). However, this pattern of conservation was not detected in the homologous regions of chromosomes 1 and 3 in genome A (Fig. 2E and Table S10). Combined with the absence of chromosomal fragment exchange in this region, it is speculated that this phenomenon is due to the high conservation of the *ASMT/COMT* gene cluster itself rather than the reorganization of genome structure. In contrast, the homology distribution of chromosomes 4, 5, and 7 is highly consistent with known fragment exchange events [47, 48]. Considering the *ASMT/COMT* gene family’s long evolutionary history [26], and its coevolution with genome structural remodeling is plausible (Fig. 2E and Table S10). Within homologous subgenomes, *ASMT/COMT* genes exhibited highly conserved syntenic patterns across all chromosomes except chromosome 5, which showed weaker syntenic relationships (Fig. 2D). Although this observation supports that the *ASMT/COMT* gene family expanded through WGD. Consequently, we traced and statistically analyzed the evolutionary origins of duplication events for each gene (Fig. 2F). Regarding the number of *ASMT/COMT* copies, the A genome basically fluctuates at ~30, while the B genome and the D genome are ~45 (Fig. 2F). The copy numbers of *ASMT/COMT* in different ploidy wheat from the bar chart can further verify the retention phenomenon of *ASMT/COMT* obtained by subchromosomal collinearity analysis (Fig. 2F). The comprehensive results showed that the *ASMT/COMT* gene family mainly expanded through WGD (56.4%), accompanied by tandem duplication to increase gene copy numbers (Fig. 2F and Table S11).

### Comparison of *ASMT/COMT* CNV in the allopolyploid plant wheat and *Ae. tauschii* at the pan-genome level

**Figure 3:**
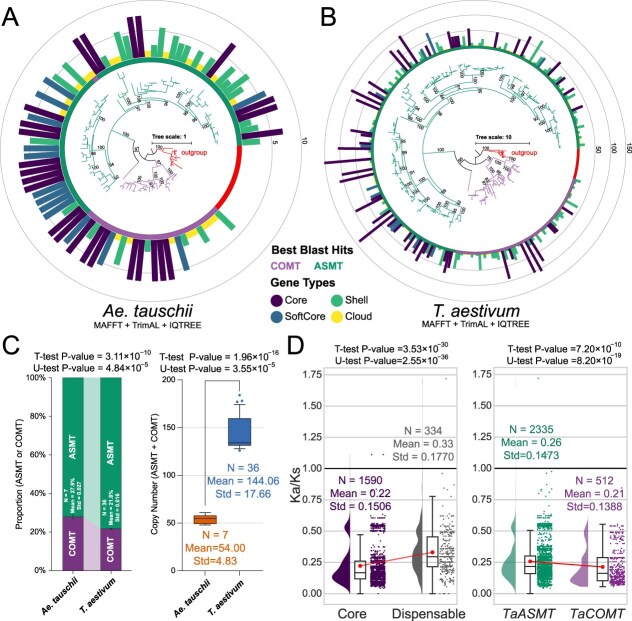
Comparison analysis of *ASMT/COMT* CNV and selection pressures between *Ae. tauschii* and wheat. **(A)** Phylogenetic tree of *AetASMT/AetCOMT* OGGs identified in the *Ae. tauschii* pan-genome. The unrooted maximum-likelihood tree was constructed using IQ-TREE v2.4.0. *Marchantia polymorpha*’s ASMT sequence was used as the outgroup to calibrate the roots. The bar chart shows the corresponding number of genes in OGGs and distinguishes different types with colors, including Core (the proportion of varieties included = 100%), Shell (the proportion of varieties included >90%), SoftCore (90% ≤ the proportion of varieties included ≤10%), and Cloud (the proportion of varieties included <10%). **(B)** Phylogenetic tree of *TaASMT/TaCOMT* OGGs identified in the wheat pan-genome. **(C)** Distribution of *ASMT/COMT* CNV in *Ae. tauschii* and wheat. **(D)** Comparison analysis of selection pressures between different categories (Core and Dispensable genes at wheat pangenome level, *TaASMT*, and *TaCOMT*).

After pan-genome-wide gene family identification, 247 candidate wheat *TaASMT/TaCOMT* orthologous gene groups (OGGs) (comprising 5186 genes from the wheat pan-genome, which included 36 wheat varieties) and 86 candidate *Ae. tauschii AetASMT/AetCOMT* OGGs (comprising 378 genes from *Ae. tauschii* pan-genome, which included seven varieties) were identified (Tables S11–S13). The distribution pattern of its gene types and the collinearity relationship demonstrate the conservation of ASMT/COMT in wheat pangenome (Fig. S2). The *TaASMT/TaCOMT* copy numbers in Hexaploid wheat (mean = 144.06) showed a 2.67-fold significant increase compared to its diploid ancestor *Ae. tauschii* (mean = 54.00) (*t*-test *P*-value = 1.96 × 10^−16^, Mann–Whitney *U* test *P*-value = 3.55 × 10^−5^) (Fig. 3A–C and Tables S11–S13). At the same time, the regularity of *ASMT/COMT* copy number obtained by large-scale identification analysis is fully consistent here. *Ae. tauschii*, species with low copies, tended to have higher COMT proportions (21.8% in wheat versus 27.9% in *Ae. tauschii*, *t*-test *P*-value = 3.11 × 10^−10^, Mann–Whitney *U* test *P*-value = 4.84 × 10^−5^) (Fig. 3C).

Selection pressure analysis (*K*a/*K*s, indication of selection pressure at protein level) revealed that the entire *ASMT/COMT* gene family underwent purifying selection, with core genes experiencing significantly stronger selective constraints than noncore genes (core genes: *K*a/*K*s = 0.22; noncore genes: *K*a/*K*s = 0.33; *t*-test *P*-value = 3.53 × 10^−30^, Mann–Whitney *U* test *P*-value = 2.55 × 10^−36^) (Fig. 3D and Table S14). Similarly, the *TaCOMT* subgroup exhibited stronger purifying selection (*K*a/*K*s = 0.21) compared to the *TaASMT* subgroup (*K*a/*K*s = 0.26, *t*-test *P*-value = 7.20 × 10^−10^, Mann–Whitney *U* test *P*-value = 8.20 × 10^−19^) (Fig. 3D and Table S14). At the same time, we can observe that there are two peaks in *K*a/*K*s of *ASMT/COMT* (Fig. 3D).

Routine gene family analyses, including motif analysis and gene structure visualization of the *TaASMT/TaCOMT* gene family using ‘Chinese Spring’ wheat as a reference, and motif analysis of the *Ae. tauschii ASMT/COMT* gene family, are presented in Figs S4 and S5. Distinct differences in motif composition exist between the *ASMT* and *COMT* subfamilies (Figs S3 and S4).

The 247 OGGs TaASMT/TaCOMT protein sequences were used to build the protein interaction network (Fig. S5A). We also conducted Gene Ontology (GO) enrichment analysis of the *TaASMT/TaCOMT* gene family members to confirm the accuracy of gene family selection using ‘Chinese Spring’ wheat as reference (Fig. S5B). Both protein–protein interaction network and GO enrichment analysis revealed that the screened gene family members were predominantly enriched in O-methyltransferase functions associated with serotonin/melatonin biosynthesis and lignin biosynthesis (Fig. S5A and B). These findings demonstrate the high accuracy of the gene family screening strategy employed in this study for identifying functionally relevant members of the *ASMT/COMT* gene family.

### Expansion pattern of *ASMT/COMT* revalidation in Solanaceae

**Figure 4:**
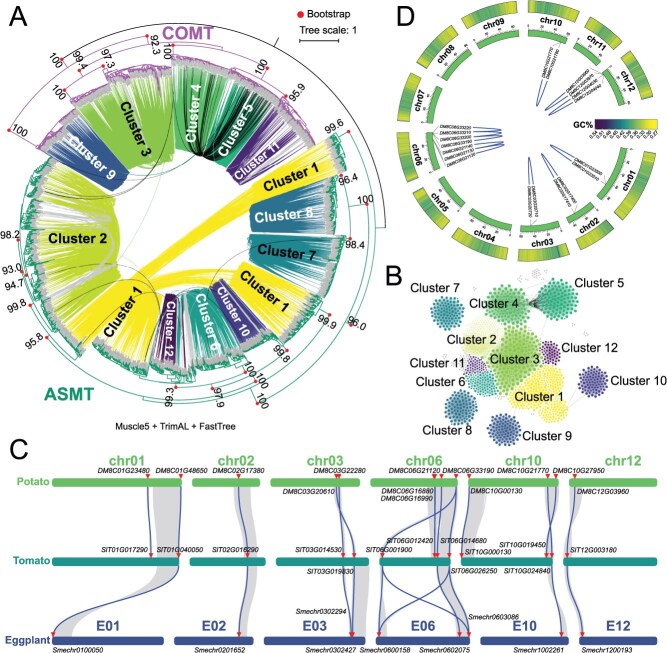
Solanaceae plants *ASMT/COMT* gene family collinearity analysis. **(A)** Comparative analysis of collinearity and phylogenetic relationships of *ASMT/COMT* genes in 88 Solanaceae species. The construction method is consistent with Fig. 1A. **(B)** Visualization of collinearity’s clusters standards of *ASMT/COMT* genes in 88 Solanaceae species. **(C)** Collinearity analysis of the *ASMT/COMT* gene family in Solanaceae plants, including potato, tomato, and eggplant. **(D)** Collinearity analysis of *StASMT/StCOMT* in *S. tuberosum* (DM genome assembly v8.1).

The *ASMT/COMT* pan-genome analysis of Poaceae and wheat species preliminarily explored the CNV pattern and the evolutionary trajectory of *ASMT/COMT*. There are still limitations in the research on the gene family expansion patterns of single-family plants, and the comparative analysis of CNV mostly focuses on allopolyploid plants. In this study, the whole-genome sequence of Solanaceae plants was utilized to deeply analyze the expansion pattern and collinearity relationship of the *ASMT/COMT* gene family (Fig. 4 and Table S15). It has laid an important foundation for the subsequent in-depth exploration of the CNV pattern of *ASMT/COMT* in the autopolyploid potato.

The phylogenetic status of the *ASMT/COMT* gene family and the collinearity network analysis further verified the highly differentiated collinearity relationship between *ASMT* and *COMT* genes (Fig. 4A and B). Specifically, the phylogenetic differences of the *ASMT* genes in Cluster 1 are significant, suggesting that this cluster may have undergone early diversification or experienced accelerated evolution (Fig. 4A). Cluster 3 and Cluster 4 exhibited not only the strongest collinearity relationship but also a close phylogenetic relationship, as they are located on the same branch on the phylogenetic tree (Fig. 4A).

Collinearity analysis was further complemented by integrating chromosome localization and collinearity block information to achieve a comprehensive analysis. The *ASMT/COMT* genes show significant collinearity on chromosomes 1, 2, 3, 6, 10, and 12 in potato, tomato (*S. lycopersicum*), and eggplant (*S. melongena*) (Fig. 4C). Combining the significance of conserved collinearity produced by tandem replication in Solanaceae plants for their adaptive evolution and integrating chromosomal localization information: on chromosome 6, the positions of *ASMT/COMT* genes are adjacent, suggesting that a tandem repeat event may have occurred. Then, we conducted a collinearity analysis on potato and initially confirmed that the expansion of the *StASMT/StCOMT* gene family within subfamilies is mainly achieved through tandem replication (Fig. 4D).

### Pan-genome level comparison of *StASMT/StCOMT* CNV in the autopolyploid plant potato

**Figure 5:**
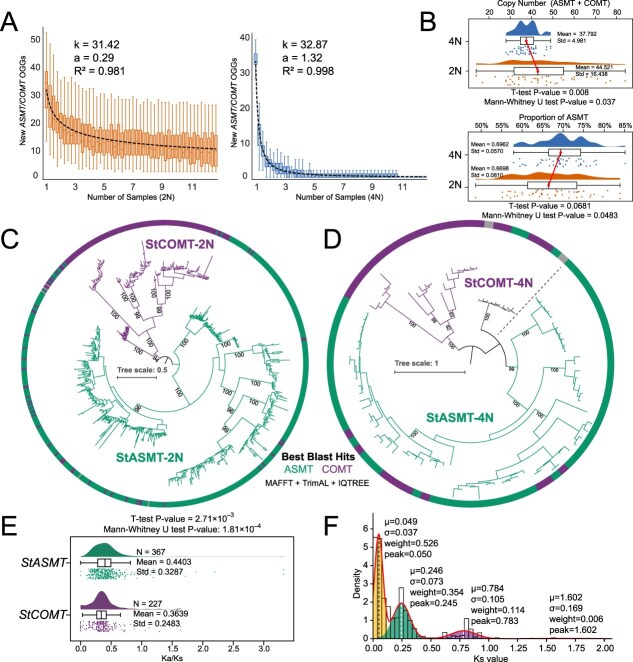
The potato pan-genome suggests the differences in the way *StASMT/StCOMT* achieve dosage balance in autopolyploid plants. **(A)** New *ASMT/COMT* OGGs curve. Convergent patterns of the *StASMT/StCOMT* gene family in diploid (left panel) and tetraploid potatoes (right panel) at the haploid pan-genome level, *n* = 100 (iterations of random sampling without replacement). Fitting of power law model of novel OGGs (*n*) and sample (*N*) with *n* = *kN^−a^*, where *k* is a constant, *−a* is the law’s exponent. *R^2^* is the Pseudo *R*-squared. **(B)** The CNV comparison of the *StASMT/StCOMT* gene family at the potato pan-genome level. From top to bottom, it respectively shows the comparison of the total copy number of the *StASMT/StCOMT* gene family and the proportion of *StASMT* subfamily members in potatoes of different ploidy. **(C)** Phylogenetic analysis of the diploid potato *StASMT/StCOMT* gene family. Infer the roots of the phylogenetic tree using the midpoint method. **(D)** Phylogenetic analysis of the tetraploid potato *StASMT/StCOMT* gene family. **(E)** Comparison of selection pressure between *StASMT/StCOMT* subfamilies (2 N and 4 N). The specific data is available in Table S17. **(F)** Peak analysis of Ks the *StASMT/StCOMT* (4 N and 2 N) gene family in potato. These peaks revealed its temporal correspondence with WGD events. Method similar to Fig. 2C.

In diploid and tetraploid potatoes, after pan-genome clustering of the *StASMT/StCOMT* gene family, the occurrence frequency of new clusters conforms to the power-law distribution, indicating that it shows convergence characteristics in the potato pan-genome, thereby significantly alleviating the potential presence/absence variations (PAVs) problem (Fig. 5A and Table S16; *k* = 31.42, *a* = 0.29, and *R^2^* = 0.981 for diploid potatoes; *k* = 32.87, *a* = 1.32, and *R^2^* = 0.998 for tetraploid potatoes). Based on phylogenetic and BLAST comparison analysis, we preliminarily identified the members of the *StASMT/StCOMT* gene family and counted their copy numbers (Fig. 5B). Analysis revealed that there were significant differences in the total copy number of *StASMT/StCOMT* genes between diploid and tetraploid haploid genomes (*t*-test *P*-value = 0.008, Mann–Whitney *U* test *P*-value = 0.037, the average value of diploid is 17.8% higher than that of tetraploid). However, the proportion of the *StASMT* gene in the total copy number did not show significant changes (Fig. 5B; *t*-test *P*-value = 0.0681, Mann–Whitney *U* test *P*-value = 0.0483). This model is significantly different from the typical allopolyploid CNV model (Fig. 3C). The phylogenetic tree structure of the *StASMT/StCOMT* genes in diploids and tetraploids shows that its clustering pattern is consistent with previous studies, distinctly divided into two branches (Fig. 5C and D). Meanwhile, the conclusion that the *StCOMT* gene is subjected to stronger negative selection pressure than *StASMT* has been further supported (Fig. 5E; *K*a/*K*s mean value of *StASMT* is 0.4403 and *K*a/*K*s mean value of *StCOMT* is 0.3639, *t*-test *P*-value = 2.71 × 10^−3^, Mann–Whitney *U* test *P*-value = 1.81 × 10^−4^). In addition, the peak value distribution of *K*s in the *StASMT/StCOMT* genes is consistent with the differentiation time point predicted based on the WGD3 and WGT1 events (Fig. 5F; the peak values of *K*s: 0.050, 0.245, and 0.783; WGD3 (GDB7) *K*s = 0.085 and WGT1 (GDB1) *K*s = ~0.7) [49]. For *StASMT/StCOMT*, we also conducted basic motif analysis and gene structure visualization (Fig. S6). The results demonstrated significant conservation within the *ASMT* and *COMT* subfamilies, both in terms of motif sequence composition (the *COMT* subgroup has specific motif sequences within the group) and gene structure (Fig. S6). In conclusion, the potato *StASMT/StCOMT* family maintains its core functions through conservative evolution and strong selection constraints during the polyploidization process.

### Analysis of the expression patterns of the *ASMT/COMT* gene family

**Figure 6:**
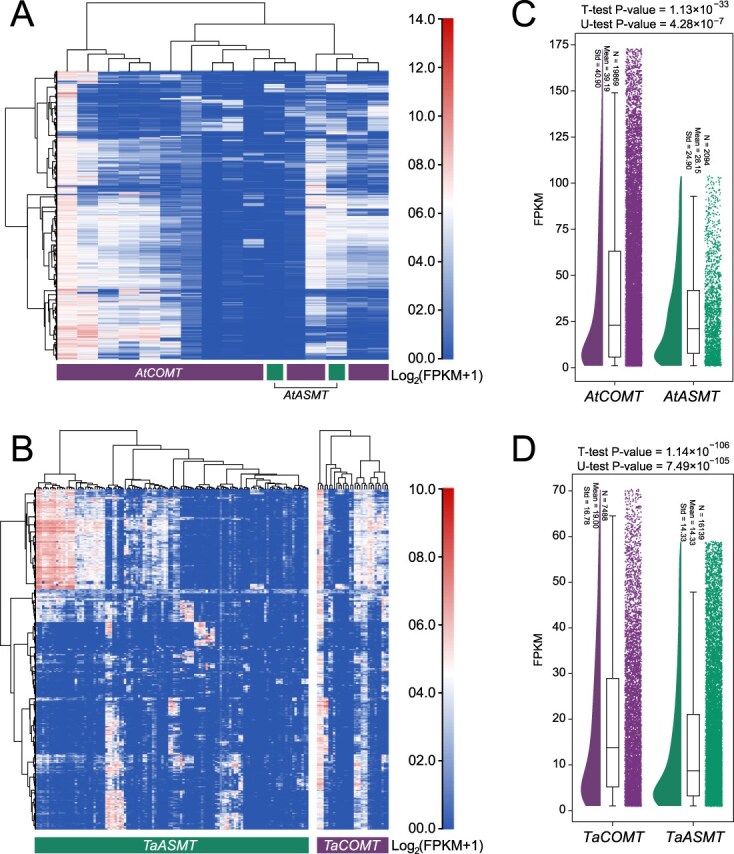
Analysis of the expression patterns of the *ASMT/COMT* gene family. **(A and B)**  *AtASMT/AtCOMT* or *TaASMT/TaCOMT* expression heatmap in *A. thaliana* (A) and wheat (B). The horizontal axis represents different genes of *ASMT/COMT*, and the vertical axis represents different transcriptomes. The categories of *AtASMT/AtCOMT* and *TaASMT/COMT* are labeled on the figure. **(C and D)** The box plots depict the overall expression differences between the *ASMT* and *COMT* gene subfamilies in *A. thaliana* (C) and wheat (D). *AtASMT/AtCOMT* and *TaASMT/TaCOMT* respectively represent all members of the subfamilies of the *ASMT* and *COMT* gene families in *A. thaliana* and wheat. For each subfamily, expression values (in FPKM) <1 were excluded from the analysis. Outliers were removed prior to plotting.

We conducted expression analyses to ensure the robustness of our evidence chain (Figs 6 and S7 and Table S18). Although RNA-seq data from >1000 experiments per species in wheat (Fig. 6A), *Arabidopsis* (Fig. 6B), and tomato (Fig. S7) revealed expression bias, previous studies have demonstrated that expression bias is an adaptive strategy in plants following WGD events. The collected transcriptome data may be more focused on plants under routine experimental conditions. And heatmap analysis indicated that most genes can be induced under specific conditions. Visually speaking, *COMT* genes in *A. thaliana* and wheat are roughly higher than that of *ASMT* (Fig. 6A and B). The biological significance obtained by using traditional gene family expression analysis methods is limited, suggesting that when analyzing the overall function of gene families from the perspective of CNV, it may be necessary to change the analysis approach. So we use boxplots to analyze the trend of *ASMT/COMT* expressions. The overall expression trend of the *ASMT/COMT* subfamily indicates that the expression levels of *COMT* genes in *A. thaliana* and wheat are both higher than that of *ASMT* (Fig. 6C and D).

## Discussion

### Horizontal gene transfer underlies the evolutionary origin of *ASMT/COMT-*mediated melatonin biosynthesis

**Figure 7:**
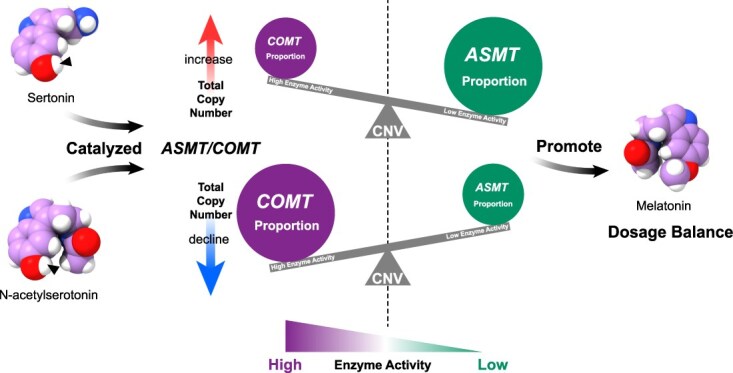
A dosage compensation model of *ASMT/COMT* CNV contributes to maintaining melatonin synthesis homeostasis. For the two functionally similar enzymes, ASMT and COMT, both can catalyze the transfer of methyl groups to the two substrates, Sertonin and N-aceylserotonin, in the melatonin pathway. We use a balance symbol marked with CNV to represent the melatonin synthesis dosage balance hypothesis proposed in this study.

HGT may provide adaptive advantages to recipient organisms, allowing them to exploit new ecological niches or resources [50, 51]. As the rate-limiting enzyme in melatonin synthesis (Fig. 7), the *ASMT/COMT* gene family originated from *ASMT* through HGT via endosymbiotic events and was integrated into the ancestral plant genome [26]. Subsequent expansion within plant lineages occurred primarily through WGD events (Fig. 2E) [26].

In Solanaceae, *ASMT* and *COMT* genes are mainly expanded via tandem duplication within subfamilies, yet their collinearity is largely driven by WGD (Fig. 4A and C). Unlike previous studies that inferred evolutionary relationships based on functional conservation or extrapolation by exclusion, our study provides the first direct evidence for the origin of *COMT* at both large-scale and pan-genome levels, thereby reinforcing and extending earlier findings. (Figs 2D, 2E, and  S1E).

At the species level, we identified 41 and 34 *ASMT/COMT* genes in soybeans and rice, respectively. Although the numbers were different from those in existing studies, such as 44 *ASMT* genes in soybeans [32] and 33 *COMT* genes in rice [21], the overall trend was consistent, reflecting that this gene family has a certain scale and species specificity in different species.

ASMT/COMT mainly catalyzes the methylation reaction (Fig. 7). Compared to *ASMT*, *COMT* acquired novel substrate catalytic activities due to its more relaxed conformation [26]. As one of the gene families that persisted throughout nearly the entire process of plant radiation evolution, the phylogenetic tree of *ASMT/COMT* exhibits distinct clustering patterns, with the species distribution of each subgroup highly consistent with plant evolutionary history. Moreover, the coincidence between the *K*s peak of the *StASMT/StCOMT* gene family in potato and the *K*s peak (Fig. 5F) attributed to the Solanaceae WGD events provides compelling molecular evidence further supporting the synchronous evolution of this gene family with the broader plant radiation process. We observed two distinct peaks in the *K*a/*K*s distribution (Fig. 3D). While two *K*s peaks are expected due to the two WGD events, the presence of two *K*a/*K*s peaks is unexpected, and its underlying cause remains unclear.

### Whole-genome duplication dynamics and purifying selection shape *ASMT/COMT* CNV

All natural plant species have evolved from ancient polyploids, and WGD events, or polyploidization, are key drivers of plant evolution [52–54]. By generating redundant genes through tetraploidization or hexaploidization, WGD promotes genetic diversity, alleviates purifying selection pressure, and facilitates mutation accumulation and functional innovation. These events preserve essential gene functions via retention mechanisms including dosage balance, subfunctionalization, and neofunctionalization, while the fractionation process preferentially removes redundant or low-expression genes, as evidenced by the rapid loss of single-copy genes in *Zea mays* and *Brassica rapa* [52]. The mechanisms underlying subgenome dominance in allopolyploids demonstrate that dominant subgenomes shape phenotypic formation by retaining dosage-sensitive genes (such as those encoding protein complex subunits or regulatory network hubs) [52, 55], while the retention or fractionation of redundant genes post-WGD is strictly constrained by stoichiometric balance, which is consistent with the dosage-sensitive synergistic effects observed in the *ASMT/COMT* gene family’s CNV pattern (Fig. 1C–F). *ASMT/COMT* is one of the subfunctionalization gene families after WGD events. Genes derived from polyploidy often undergo selective retention or loss, a process theoretically explained by the dosage balance hypothesis [52, 55, 56]. Consistent evidence from multiple analyses in the current study indicated that the *ASMT/COMT* gene family is under strong negative selection (Fig. 3D, Tables S14 and S17). This pattern of negative selection may be closely linked to the dosage-sensitive nature of *ASMT/COMT* genes and the selective constraints imposed by polyploidy-driven genome evolution.

Considering the clustering characteristics of the *ASMT/COMT* gene family (Fig. 1A), a deeper understanding of its evolutionary trajectory may require a comparative analysis of its evolutionary patterns in monocotyledonous and dicotyledonous plants. As a representative family of monocotyledons, Poaceae crops have undergone multiple WGD events [57]. Our results demonstrate that this gene family from Poaceae primarily expanded through WGD events, accompanied by a small number of tandem duplication events (Fig. 2E), a finding consistent with previous studies on *ASMT/COMT* expansion via WGD.

Taking the Solanaceae family as the representative of dicotyledonous plants, our analysis revealed significant differences in the subfamily-level expansion mechanism of *ASMT/COMT* genes between monocotyledonous and dicotyledonous plants (Figs 2E and 4C). And we speculate that the area on chromosome 6 might be a conserved collinear block formed by tandem replication of Solanaceae plants, and the *ASMT/COMT* gene family might be involved in the formation process of this conserved gene cluster (Fig. 4C). We hypothesize that the expansion mechanisms of the *ASMT* and *COMT* subfamilies differ between monocots and dicots, with support from the observation of conserved collinear blocks formed through tandem replication in Solanaceous plants [58].

It is worth noting that the collinearity relationship between *ASMT* and *COMT* in both types of plants is mainly driven by WGD events (Fig. S1B and Table S10). However, results in Fig. 4A have shown that there is not a complete absence of collinearity between *ASMT* and *COMT*, which contradicts the results in potato (Fig. 4D). Taking into account that the analysis based on a single-reference genome is difficult to solve the PAV problem of genes, it is of great significance to introduce the pan-genome for in-depth verification.

Previous studies have shown that synteny analysis can provide key information for the reconstruction of the evolutionary trajectory of gene families [59,60]. However, synteny analysis revealed significant differences in the adjacent gene composition between *ASMT* and *COMT* within the genome (Figs 2A and 4A), indicating substantial changes in their genomic microenvironments. Despite this structural divergence, the synteny-based approach precisely delineates the evolutionary divergence path of these paralogs, and sequence homology analysis showed that despite the *ASMT–COMT* differentiation event occurring in a distant evolutionary period, leading to notable variations in their genomic locations, they maintain high similarity in protein sequence structure (Table S1). This striking contrast between highly conserved protein sequences and significantly divergent genomic contexts further highlights the importance of the *ASMT/COMT* gene family in functional evolution.

### *ASMT/COMT* CNV driving melatonin dosage balance

As a gene family expanded by WGD and under strong negative selection pressure, we propose that the evolutionary pattern of the *ASMT/COMT* gene family aligns with the dosage balance hypothesis. Based on our CNV data (Fig. 1C, E, and  F) and previous research indicating that *COMT* evolved from *ASMT* with enhanced catalytic activity [26], we propose a complementary ‘efficiency–dosage’ mechanism. We hypothesize that in low-copy species, the abundance of *COMT* compensates for the copy number limitation by leveraging its inherently higher catalytic efficiency. Conversely, in high-copy species, *ASMT* may achieve precise metabolic regulation through gene dosage amplification. This ‘efficiency–dosage’ complementary mechanism not only retains the regulatory characteristics of *ASMT* as an ancestral gene but also gives full play to the catalytic potential of *COMT* to maintain the dynamic balance of the melatonin metabolic network (Fig. 7).

Typically, gene families with high copy numbers are more susceptible to sequence divergence, enabling neofunctionalization or subfunctionalization. The potential evolutionary mechanisms that allow the *ASMT/COMT* family to maintain high sequence conservation despite its extensive copy number expansion remain unclear. The divergence times for the *ASMT/COMT* family across grass species is 26 Mya. From an evolutionary perspective, this is a relatively recent event. We speculate that the expansion and divergence of the *ASMT/COMT* family are indeed evolutionarily recent. The limited time since duplication has likely prevented widespread sequence divergence or pseudogenization, providing a parsimonious explanation for the high sequence identity we observe.

For phytohormones, factors that affect the sensitivity of phytohormones include variations in the number of receptors, differences in the binding capacity of receptors, and the type of downstream event in response. Traditionally, dosage balance studies have focused on the coregulation of protein complex subunits. Since the dosage balance hypothesis explains dosage-sensitive regulation, and phytohormone homeostasis similarly depends on receptor-mediated sensitivity, we applied this framework as an indirect validation strategy. Therefore, in view of the limitations of quantitative data for melatonin in multiple species, we further performed indirect validation from the perspective of substrate–receptor dosage balance (Fig. 1E and F). The number of melatonin receptors does not change significantly due to the alteration in the number of *ASMT/COMT* copies, so this result implies that melatonin content was stable at the physiological requirement threshold and not directly affected by *ASMT/COMT*’s CNV (Fig. 1E and F).

Furthermore, to investigate the differences in WGD mechanisms, we selected potato, a solanaceous plant with a complete genome, as a representative autopolyploid and compared it with the allopolyploid wheat. Our comparative analysis led us to preliminarily infer that the gene dosage balance of potato’s *ASMT/COMT* genes is achieved primarily through changes in total copy number, rather than by regulating the proportion of specific subfamilies. We speculate that this is due to the high similarity of homologous chromosomes and gene loss during potato polyploidization, suggesting that dosage balance does not solely rely on subfamily proportion. Previous studies have shown that the high recombination rate promotes the rapid loss of redundant genes through DNA deletion, especially in the early stage of polyploidy [61]. Therefore, the reason for this might be the high similarity of homologous polyploid genomes and the loss of genes during the process of potato polyploidization. We initially infer that the achievement of dosage balance does not solely and directly rely on the regulation of the proportion of subfamilies.

Notably, the gene dosage balance hypothesis not only acts on the balance of expression among gene copies but also on the differential regulation of expression patterns of homologous genes after WGD events. This suggests that gene dosage balance can be achieved by both CNV and expression bias adjustment, ultimately contributing to the maintenance of functional product homeostasis. Based on our model, to maintain the dosage balance of metabolic pathways, the expression patterns of the two should tend to be consistent. Although this phenomenon may seem inconsistent with the principle of dosage balance on the surface (Fig. 6), in fact, COMT not only participates in the synthesis of melatonin but also widely engages in other pathways such as lignin synthesis. Its relatively high expression level may precisely be to meet the requirements of the coordinated regulation of multiple metabolic pathways, thereby achieving functional balance at the system level.

A key consideration in interpreting our evolutionary analyses is the well-documented broad substrate specificity of COMT, which is involved in multiple pathways such as lignin biosynthesis. Studies outline the two parallel biosynthetic routes for melatonin, one involving ASMT and the other involving COMT, treating them as comparable enzymatic options for the final O-methylation step [62]. Functional evidence demonstrated that COMT possesses inherent ASMT activity and that its overexpression enhances melatonin production and stress tolerance [63, 64]. Crucially, previous studies explicitly conducted a joint evolutionary analysis of *ASMT* and *COMT*; thus, the joint analysis in our research is reasonable. Moreover, the comparison between species in the low- and high-copy number groups is effective because some seminal studies [65, 66] employed gene copy number as a primary metric to demonstrate convergent reduction of immune receptor repertoires in plants adapting to special lifestyles, directly linking CNV to ecological function and selective pressure. This validates CNV analysis as a legitimate initial approach for inferring functional adaptation.

Research shows that the evolution of enzymes is essentially about seeking a balance among catalytic efficiency, cellular metabolic demands, and resource allocation [67]. Based on this, the evolution of the *ASMT/COMT* gene family is very likely guided by its metabolites. This view of this study has been supported by multiple pieces of evidence: the copy number of the *Mla7* gene in wheat is positively correlated with the disease resistance level [68]; in addition, *CIPK* and *CBL* achieve dosage balance through expression regulation [6], which also demonstrates the influence of expression levels on copy number variations. The above-mentioned phenomenon precisely reflects the specific evolutionary strategies adopted at different levels during the evolution of enzymes to achieve a balance among function, demand, and resources, thereby strongly supporting the rationality of this study.

### Research limitations and introspection

We acknowledged that there are limitations in species representation within current whole-genome assemblies, and the current classification system of ASMT and COMT is mainly based on phylogenetic status and sequence similarity. The overall classification framework is basically consistent with the trend of gene function evolution, but the functional definition of subfamily boundaries still needs to be verified by experiments. The comparison of allopolyploid wheat and autotetraploid potato is limited, as only one species from each group is included. This raises reliability concerns, since differences may be confounded by other factors such as monocot and dicot divergence, which is also known to influence gene family expansion. Notably, the phenotypic consequences of ASMT/COMT dosage imbalance on stress tolerance remain unverified, as neither gene knockout nor overexpression assays have been implemented. First, leveraging natural CNV in wheat and potato germplasm to test dosage effects; second, applying targeted mutagenesis and transgenic overexpression to engineer dosage-imbalanced lines, thereby probing CNV–metabolic homeostasis interactions; third, extending the combined research paradigm of ‘evolutionary analysis–function verification’ established in this study to WGD or other types of gene replication extended and subfunctionalized gene families such as the cytochrome P450 superfamily [69]. This integrated framework will advance dissection of dosage balance in complex trait regulation and inform polyploid crop breeding strategies.

## Materials and methods

### Acquisition of genomic data

The genomic data of 1117 species were downloaded from the plant genome database PlantGIR (http://plantgir.cn/) [70], including protein sequences, coding sequences, and genome annotation files in GFF format (gene ID, chromosome number, start, stop, strand, and gene ID). After strict quality control screening, the complete dataset of 1052 species was finally selected for the evolutionary analysis of *ASMT/COMT* gene family.

For pan-genomic data integration, a multisource data fusion strategy was used: wheat (*T. aestivum*) genomic data were collected from CNCB (https://www.cncb.ac.cn/) and Ensembl Plants database (https://plants.ensembl.org/) [37, 71–73]. The *Ae. tauschii* genomic data, including *Ae. tauschii* TA10171, TA1675, TA2576, T093, XJ02 AY61, and AY17 genomes, was downloaded from China National GeneBank (CNGB) (https://db.cngb.org/search/project/CNP0001325/) and GrainGenes online database (https://wheat.pw.usda.gov/GG3pangenome/wheat/D/taus_home.php) [74, 75]. The diploid and tetraploid potato genomic data was available on the Potato Repositories Website (http://solomics.agis.org.cn/potato/) (46 genomes of wild diploid potato) [36, 76], planT2T genomic database (https://bis.zju.edu.cn/plant2t/home) (the haplotype-resolved T2T genome of *S. commersonii*) [58], and Zenodo [77] (https://doi.org/10.5281/zenodo.14053896) (11 genomes of tetraploid *S. tuberosum*) [41]. *Arabidopsis thaliana* pan-genome data [38, 78] were obtained from figshare [38] (https://doi.org/10.6084/m9.figshare.21673895.v1 or https://figshare.com/articles/dataset/32_ecotypes_Arabidopsis_thaliana_genomes_gene_annotation_pan-TE_library_graph_pan-genome_gene_family_and_gene_presence_absence_matrices_files_/21673895).

All genome files were formatted and standardized with the use of TBtools v2.225 to ensure compatibility for subsequent analysis [79].

### Large-scale identifications of *ASMT/COMT* and *CAND2/PMTR1* genes

To systematically identify *ASMT/COMT* genes, comprehensive genome scanning of ASMT/COMT was performed using HMMER 3.3.2 [80], DIAMOND v2.1.9.163 [81] based on the hidden Markov model profile of ASMT/COMT (PF08100, PF00891) downloading from interPro (https://ftp.ebi.ac.uk/pub/databases/Pfam/releases/Pfam35.0/) [82] and the protein sequence of ASMT/COMT downloading from UniPort (https://www.uniprot.org/). The *E*-value is 1.0 × 10^−5^ for both hmmerscan and BLASTP. And the protein sequence length was limited to 200–600 aa to exclude abnormal sequences caused by gene annotation errors (>600 aa may be caused by tandem duplication, <200 aa has the risk of functional domain loss). To characterize potential protein structural variations within the *ASMT/COMT* gene family, we conducted motif analysis using the MEME tool on *ASMT/COMT* proteins identified [83].

To systematically identify *CAND/PMTR1* genes, the hidden Markov model profile of CAND/PMTR1 (PF10160) and *A. thaliana* CAND/PMTR1 protein sequence (Q94AH1, CAND2_ARATH) from UniPort were used for hmmscan and BLASTP. The *E*-value is 1.0 × 10^−5^ for both hmmerscan and BLASTP.

### Inference of orthologous gene groups in pan-genome-wide gene family

Based on the previously established pan-genome gene family analysis pipeline [84], CD-HIT v4.8.1 [85] was used (Sequence identity ≥95%, alignment coverage ≥90%) to get protein sequences clusters, and the longest protein sequence in each cluster was used as the representative sequence of each orthologous gene group in wheat, *Arabidopsis*, *Ae. tauschii*, and potato pangenomes. Based on the PAV of OGGs in different varieties, we classified the genes into four categories including Core (the proportion of varieties included = 100%), Shell (90% ≤ the proportion of varieties included <100%), Softcore (10% ≤ the proportion of varieties included <90%), and Cloud (the proportion of varieties included <10%). And the dispensable ASMT/COMT represents OGGs absent in at least one variety.

### Phylogenetic tree construction

For large-scale identification result, we used muscle v5.3 in Super5 algorithm for the excessive number of ASMT/COMT protein sequences to align the sequence [86]. TrimAL v1.5 was used to delete nonconserved amino acid in gappyout model [87]. And sequences >50% with gaps were deleted to avoid the attraction of phylogenetic trees. Finally, these sequences were used to build the phylogenetic tree through FastTree v2.1.11 in “LG” model [88, 89]. Chlorophyta and Bryophyta branches were used as outgroup. For pan-genome-wide identification result, MAFFT v7.525 was used to align the sequence in “auto” mode [90]. And TrimAL v1.5 was used to delete nonconserved amino acid in ‘automated1’ model. The unrooted maximum-like-lihood tree was constructed using IQ-TREE v2.4.0 [91] (1000 UltraFast Bootstrap [92] evaluation of node support, ModelFinder preferred the best alternative model for each dataset). *Marchantia polymorpha*’s ASMT sequence was used as the outgroup to calibrate the roots. The specific methods will be detailedly marked under the annotations of each phylogenetic tree. And MEME (min-width: 6, min-width: 50) was used to analyze the conserved motifs of ASMT/COMT [83].

### Identification of the key loci driving the evolutionary divergence of *ASMT/COMT*

Based on the classification results of *ASMT* and *COMT*, 26207 ASMT/COMT protein sequences were used·for·training to find the key loci driving the evolutionary divergence of ASMT/COMT through random forest [44] and XGBoost [45]. The relevant code can be obtained on figshare (https://doi.org/10.6084/m9.figshare.30315934.v2 or https://figshare.com/articles/dataset/Large-Scale_Plant_Genomic_Identification_and_Analysis_Uncover_i_ASMT_COMT_i_Copy_Number_Variation_Driving_Melatonin_Dosage_Balance/30315934).

### Analysis of gene duplication and synteny

In this study, an automated analysis pipeline was constructed to analyze the genomic collinearity. First, DIAMOND v2.1.9.163 [81] was integrated into Shell script to perform an efficient alignment (*E*-value ≤1.0 × 10^−5^) of the whole genome protein sequences of 104 Poaceae species or 88 Solanaceae species, which significantly improved the efficiency of large-scale data analysis. Subsequently, MCScanX v1.0 [93] was used to analyze the pairwise alignment results in depth to systematically identify cross-species conserved gene blocks and reconstruct their evolutionary trajectories. And the cluster of synteny network was determined through Gephi [94] v0.10.1’s modularity class algorithm. Rapid genome-wide collinearity analysis of all the pangenomes was performed using TBtools v2.225 [79] integrated MCScanX plugin (One step MCScanX superfast): high-speed alignment of protein sequences (*E*-value ≤1.0 × 10^−5^) was completed based on DIAMOND v2.1.9.163, and the collinearity relationship within the genome was analyzed in one step by combining chromosome position information with MCScanX algorithm. The automated identification and classification statistics of segmental and tandem duplication events were implemented simultaneously, and the types of gene duplication were finally determined.

### Analysis of natural selection

To calculate pairwise gene *K*a, *K*s, and *K*a/*K*s values, homologous gene pairs identified through collinearity analysis were analyzed using the *K*a, *K*s, *K*a/*K*s Calculator plugin in TBtools (Simple *K*a/*K*s Calculator) [79]. Gene pairs with sequence coverage <75% were excluded from the *K*s peak analysis to ensure alignment quality. Regarding the wheat data in Fig. 3D, we specifically reexamined the 39 pairs with lower coverage. The analysis confirmed that the low coverage was generally due to genuinely shorter open reading frames in the homologs, rather than alignment issues. Given that these 39 pairs represent only a small fraction of the total 2932 gene pairs analyzed, and their limited number is unlikely to substantially influence the overall results or conclusions, we have retained them for a comprehensive analysis. The *K*s distribution was decomposed using Gaussian Mixture Models, with the optimal number of components determined by the Bayesian Information Criterion to identify *K*s peaks corresponding to WGD events from different evolutionary periods. The divergence time was estimated using the formula *T* = [*K*s/(2 × 6.5 × 10^−9^)] × 10^−6^ (million years) [95].

### Transcriptome analysis of *ASMT/COMT* genes

Expression levels of *ASMT/COMT* genes in both wheat and *A. thaliana* were obtained in fragments per kilobase per million reads (FPKM) from PPRD (http://ipf.sustc.edu.cn/pub/plantrna/) [96]. The expression levels (FPKM) of the *ASMT/COMT* gene family in tomato were downloaded from plantExp (https://biotec.njau.edu.cn/plantExp/) [97].

### Protein interaction network construction and GO enrichment analysis

The ASMT/COMT protein interaction analysis in wheat was performed using the STRING database (https://cn.string-db.org) with a high confidence score of 0.70. And the GO enrichment analysis in the wheat *ASMT/COMT* gene family was done through agriGO (https://systemsbiology.cau.edu.cn/agriGOv2/) [98,99].

### The ASMT/COMT protein structure prediction

AlphaFold3 [100] was used to predict the protein structure (https://deepmind.google/technologies/alphafold/alphafold-server/).

### Data plotting and statistical analysis

Gene expression heatmaps were generated using TBtools v2.225 [79], and network topologies were constructed and visualized in Gephi v0.10.1 [94]. The auxiliary charts were implemented by Python. The key mathematical verification was completed through Python’s SciPy scientific computing library [101], and the core analysis code has been completely archived for reproduction. For phylogenetic trees, iTOL (https://itol.embl.de/) [102] was used for visualization. For protein 3D structures, PyMOL v2.6 (https://pymol.org/) was used for visualization.

## Supplementary Material

Web_Material_uhaf348

## Data Availability

All data supporting the findings of this study is available in the article and the Supplementary information files. All source codes and raw data can be obtained on figshare (https://doi.org/10.6084/m9.figshare.30315934.v2 or https://figshare.com/articles/dataset/Large-Scale_Plant_Genomic_Identification_and_Analysis_Uncover_i_ASMT_COMT_i_Copy_Number_Variation_Driving_Melatonin_Dosage_Balance/30315934).
