# Supplementary material for: Large-scale plant genomic identification and analysis uncover ASMT/COMT copy number variation driving melatonin dosage balance
Source: Hortic Res. 2025 Dec 18;13(3):uhaf348. doi: 10.1093/hr/uhaf348 (PMC13002329; doi:10.1093/hr/uhaf348)
Supplement: Web_Material_uhaf348 [file web_material_uhaf348.zip › Figure_S4.pdf]

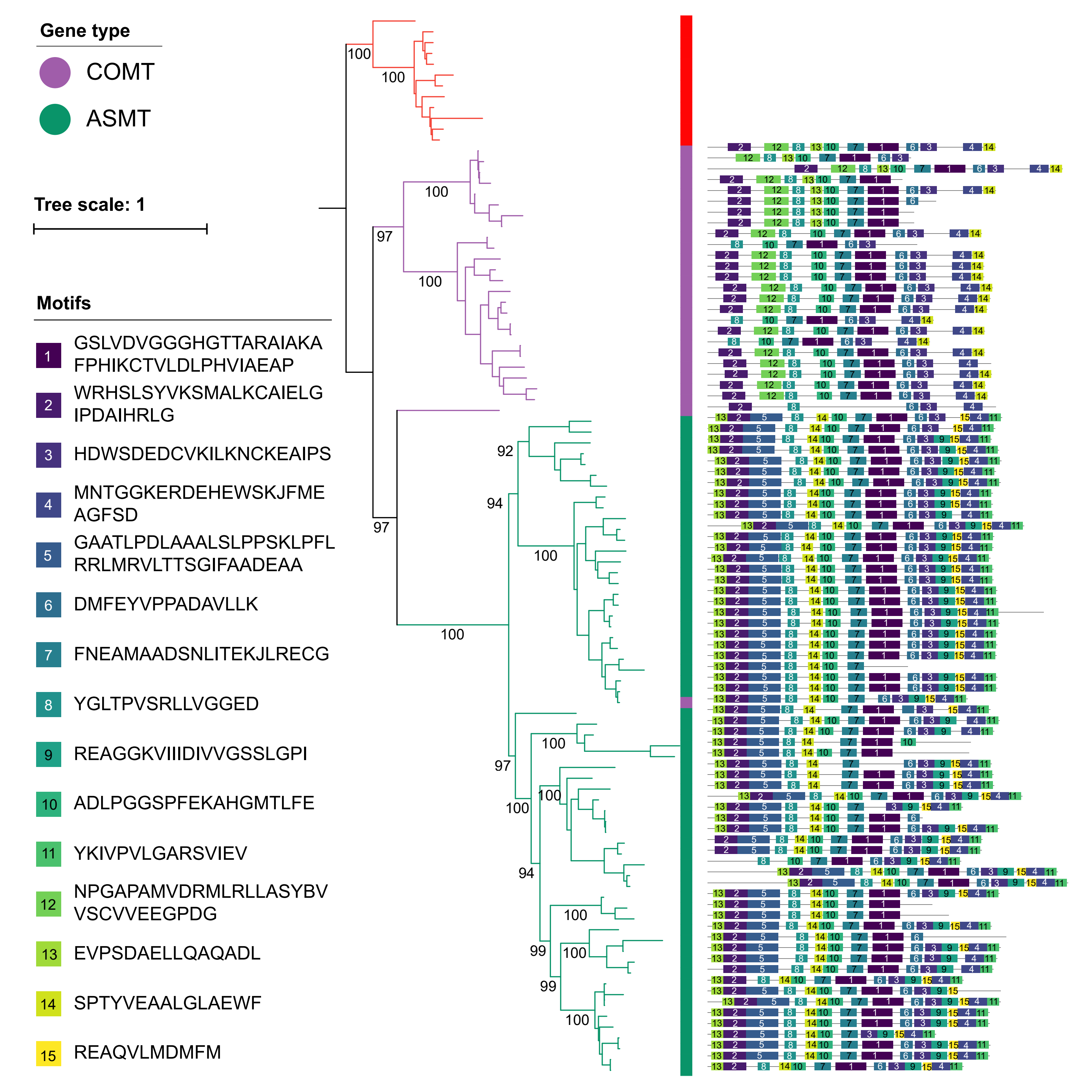
Gene type
COMT
ASMT
Tree scale: 1
Motifs
1 GSLVDVGGGHGTTARAIAKA FPHIKCTVLDLPHVIAEAP
2 WRHSLSYVKSMALKCAIELG IPDAIHRLG
3 HDWSDEDCVKILKNCKEAIPS
4 MNTGGKERDEHEWSKJFME AGFSD
5 GAATLPDLAAALSLPPSKLPFL RRLMRVLTTSGIFAADEAA
6 DMFEYVPPADAVLLK
7 FNEAMAADSNLITEKJLRECG
8 YGLTPVSRLLVGGED
9 REAGGKVIIIDIVVGSSLGPI
10 ADLPGGSPFEKAHGMTLFE
11 YKIVPVLGARSVIEV
12 NPGAPAMVDRMLRLLASYBV VSCVVEEGPDG
13 EVPSDAELLQAQADL
14 SPTYVEAALGLAEWF
15 REAQVLMDMFM
