# Supplementary material for: Large-scale plant genomic identification and analysis uncover ASMT/COMT copy number variation driving melatonin dosage balance
Source: Hortic Res. 2025 Dec 18;13(3):uhaf348. doi: 10.1093/hr/uhaf348 (PMC13002329; doi:10.1093/hr/uhaf348)
Supplement: Web_Material_uhaf348 [file web_material_uhaf348.zip › Supplementary_Material.docx]

Large-Scale Plant Genomic Identification and Analysis Uncover *ASMT/COMT* Copy Number Variation Driving Melatonin Dosage Balance

Shuotong Liu (刘烁曈)^1^, Pei Yu (于培)^1*^

^1^ SDU-ANU Joint Science College, Shandong University, Weihai 264209, China

^*^ Correspondence: [yupei@sdu.edu.cn](mailto:yupei@sdu.edu.cn)

**Supplementary Figures**

**
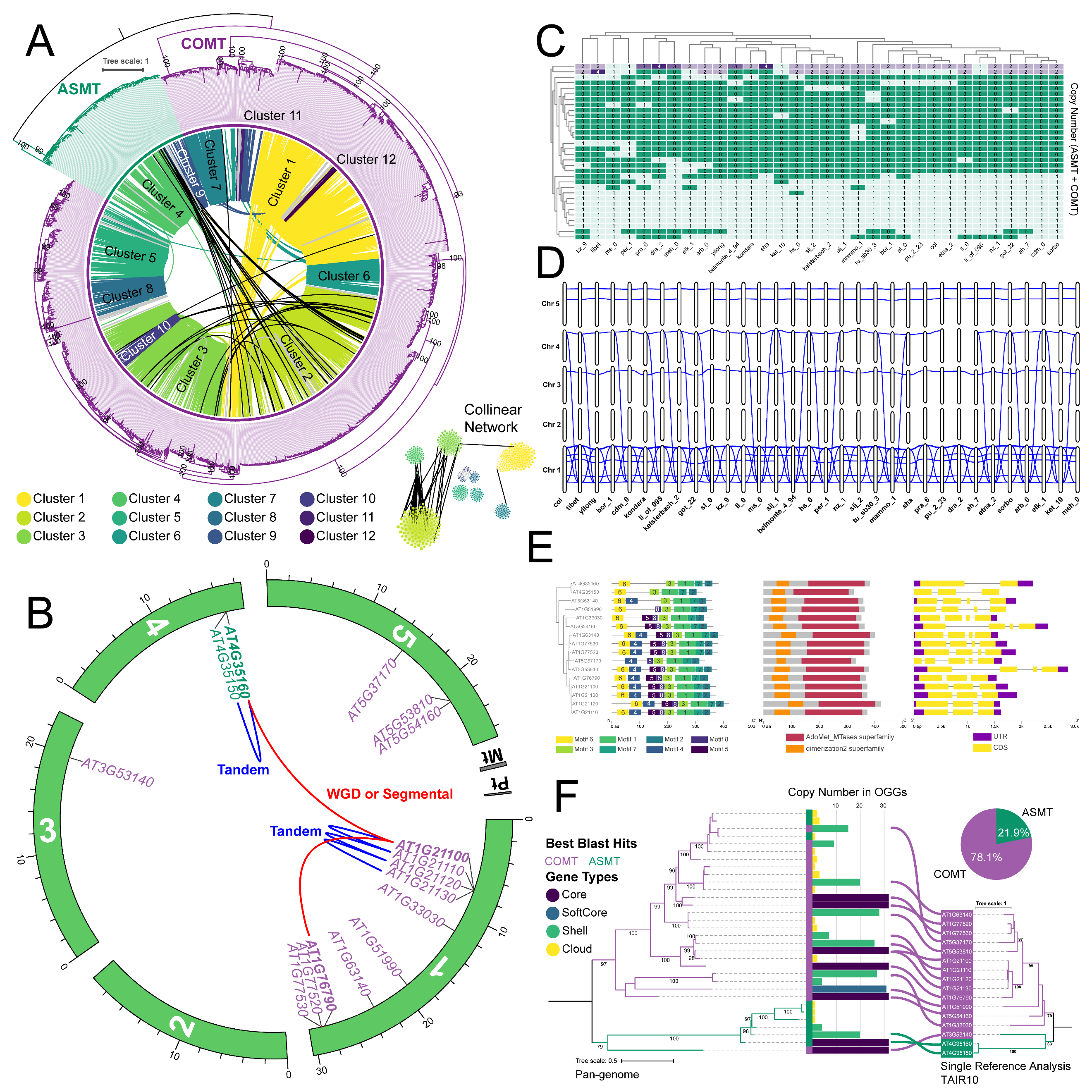
**

**Figure S1. The *ASMT/COMT* gene family in Brassicaceae plants and the *A. thaliana* pan-genome.**

**(A)** Comparative analysis of collinearity and phylogenetic relationships of *ASMT/COMT* genes in Brassicaceae plants. Based on the collinear network, we use Gephi to cluster and group *ASMT/COMT*. And the clustering results were compared and analyzed with the phylogenetic relationship. Among them, the phylogenetic tree infers the root through the midpoint method. The key nodes in the figure are marked with Bootstrap values.

**(B)** Collinearity analysis of *AtASMT/AtCOMT* in *A. thaliana*.

**(C)** Heat map of presence/absence variants (PAV) in orthologs of the *AtASMT/AtCOMT* gene family in *A. thaliana*. Each column represents the chromosome corresponding to a variety.

**(D)** Collinearity analysis of the *AtASMT/AtCOMT* gene family in the *Arabidopsis* pan-genome.

**(E)** Motif, protein domain and gene structure analysis of *AtASMT/AtCOMT* gene family in *A. thaliana*.

**(F)** Comparison of the Arabidopsis *AtASMT/AtCOMT* gene family pan-genome with a single reference. The left side is pan-genome analysis and the right is single reference analysis. The connection lines are used to represent the relationship between a single reference gene and the pan-genome OGGs.

**
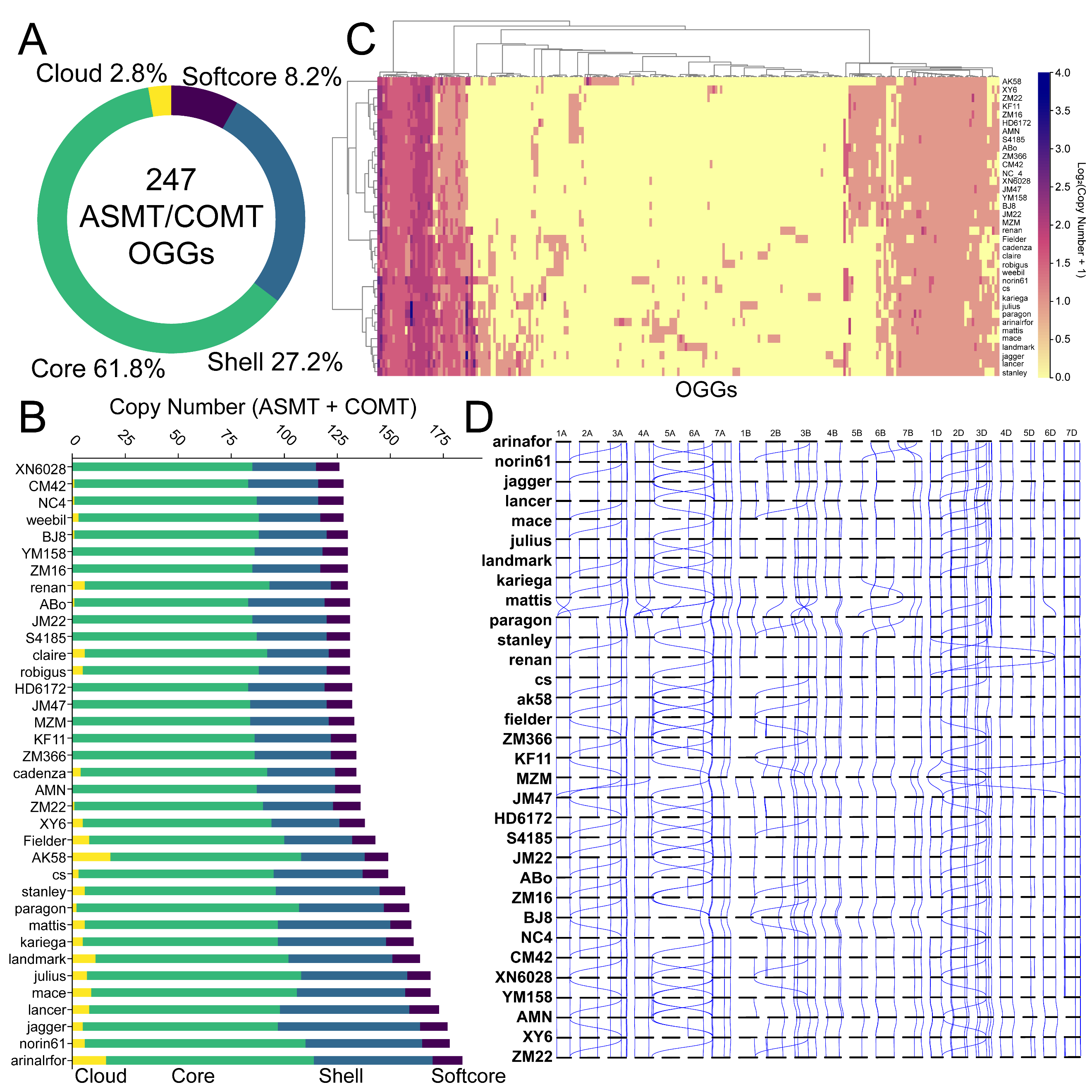
**

**Figure S2. The *TaASMT*/*TaCOMT* gene families in the wheat pan-genome.**

**(A)** The pie chart of gene type distribution in wheat pangenome of the *TaASMT/TaCOMT* gene family.

**(B)** Pattern of gene type distribution in wheat pangenome of the *TaASMT/TaCOMT* gene family.

**(C)** Heat map of presence/absence variants (PAV) in orthologs of the *TaASMT/TaCOMT* gene family in Wheat.

**(D)** Collinearity analysis of the *TaASMT/TaCOMT* gene family in the Wheat pan-genome.


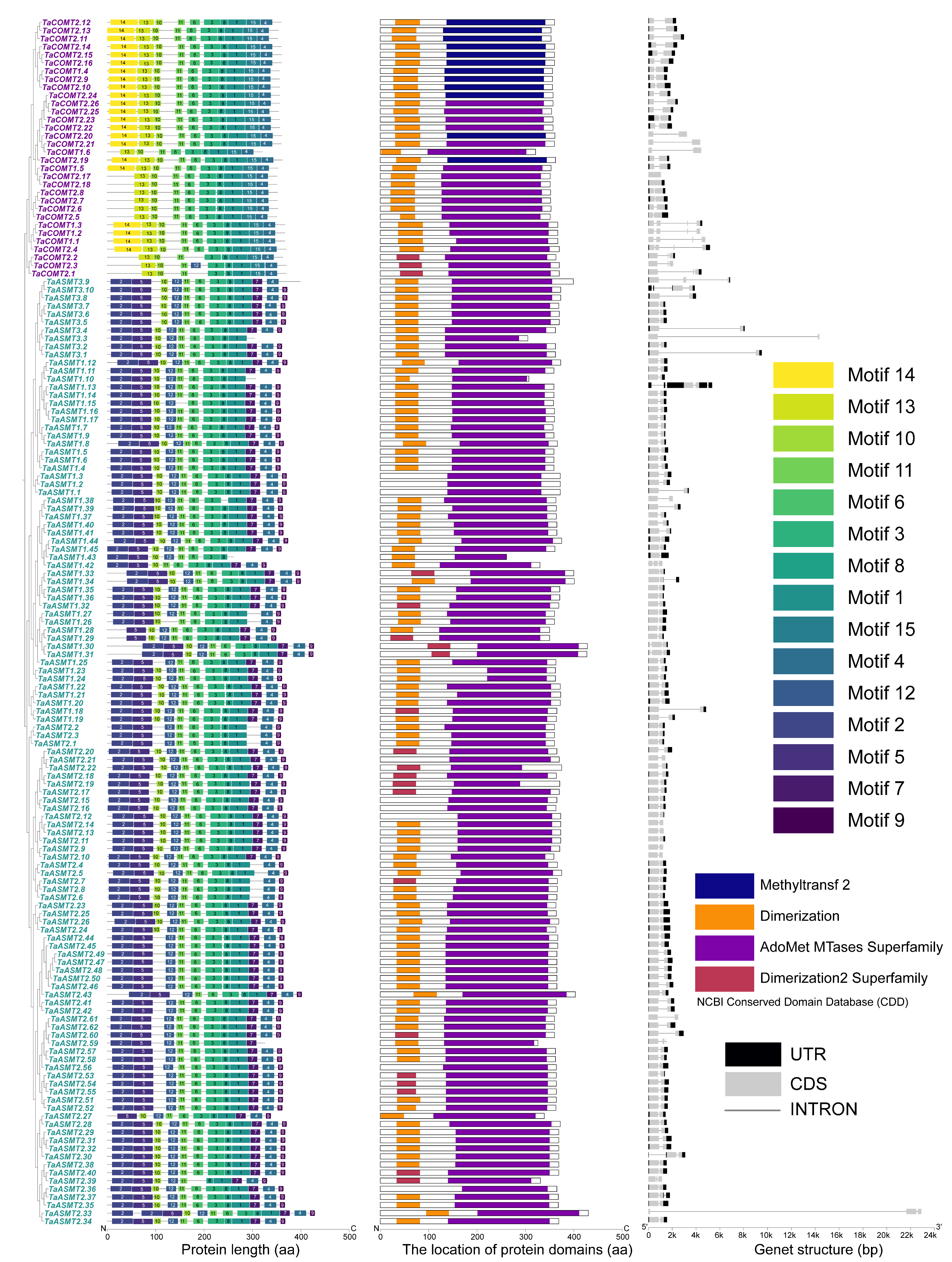


**Figure S3. Motif, protein domain and gene structure analysis of the *TaASMT/TaCOMT* gene family in Wheat.** The table of renamed IDs and the corresponding original IDs is in Table S19.


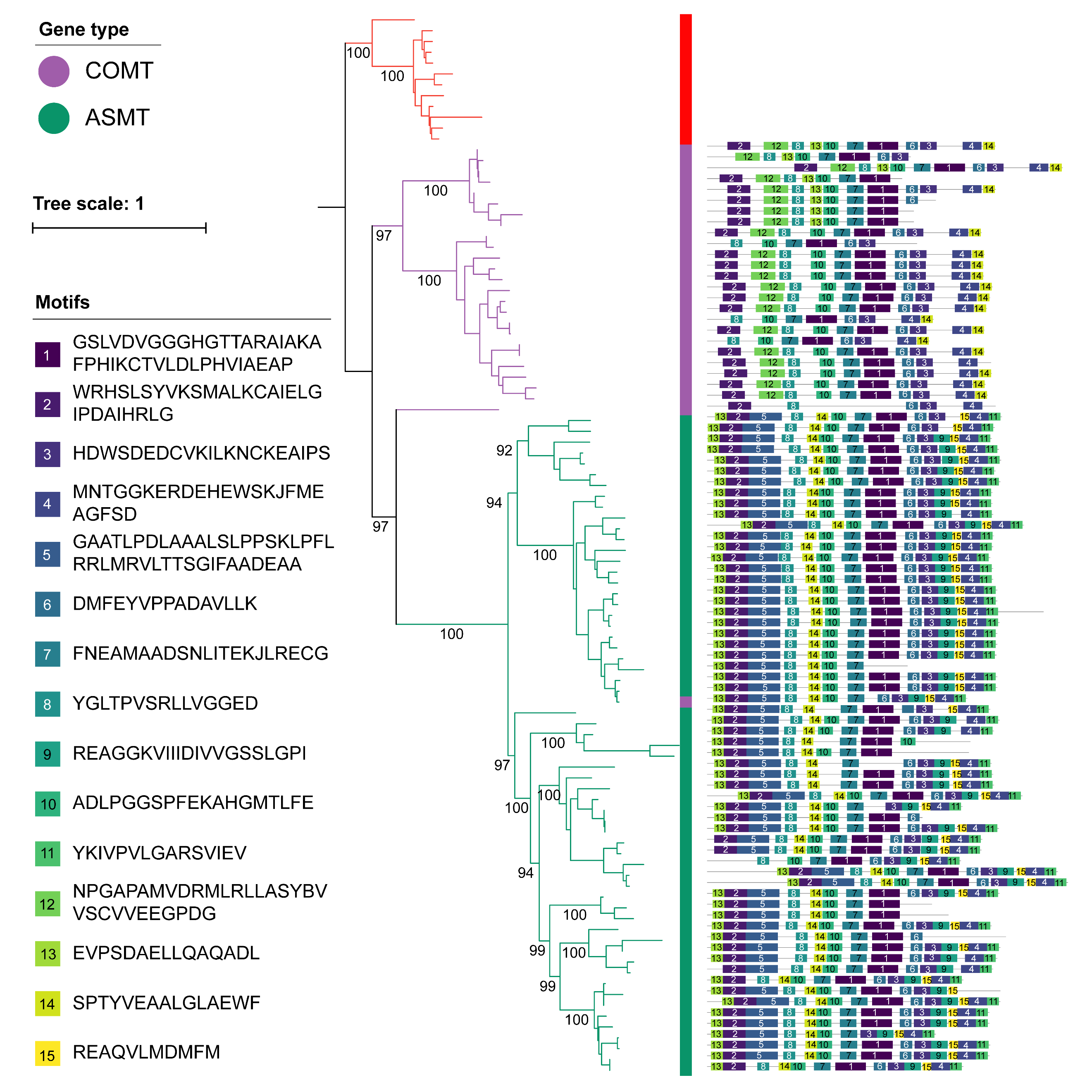


**Figure S4. Phylogenetic and motif analysis of the *AetASMT/AetCOMT* gene family in the pangenome of *Ae. tauschii*.**


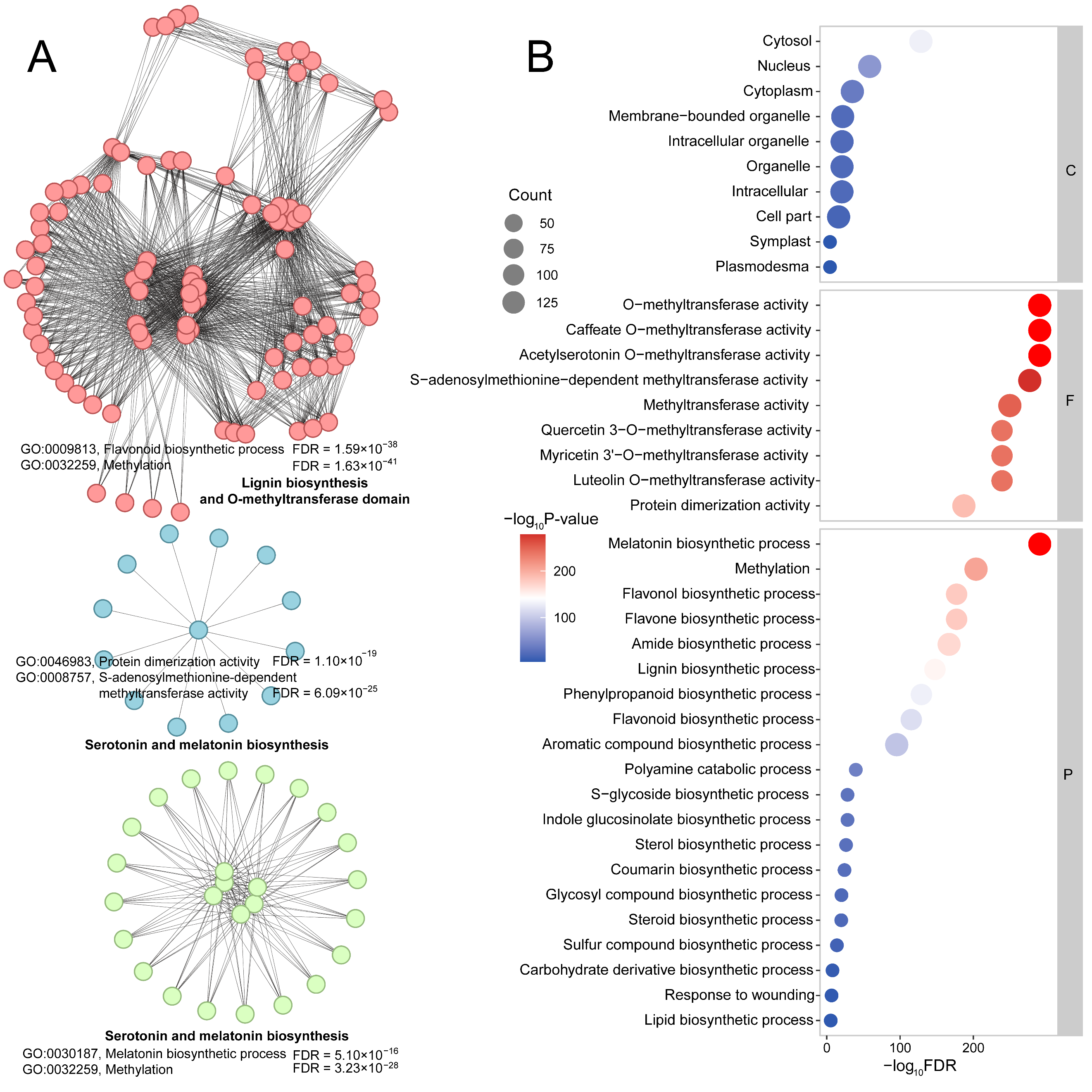


**Figure S5. Revalidation of identification results of the *TaASMT/TaCOMT* gene family in wheat.**

**(A)** TaASMT/TaCOMT protein interaction network.

**(B)** *TaASMT/TaCOMT* GO enrichment analysis.


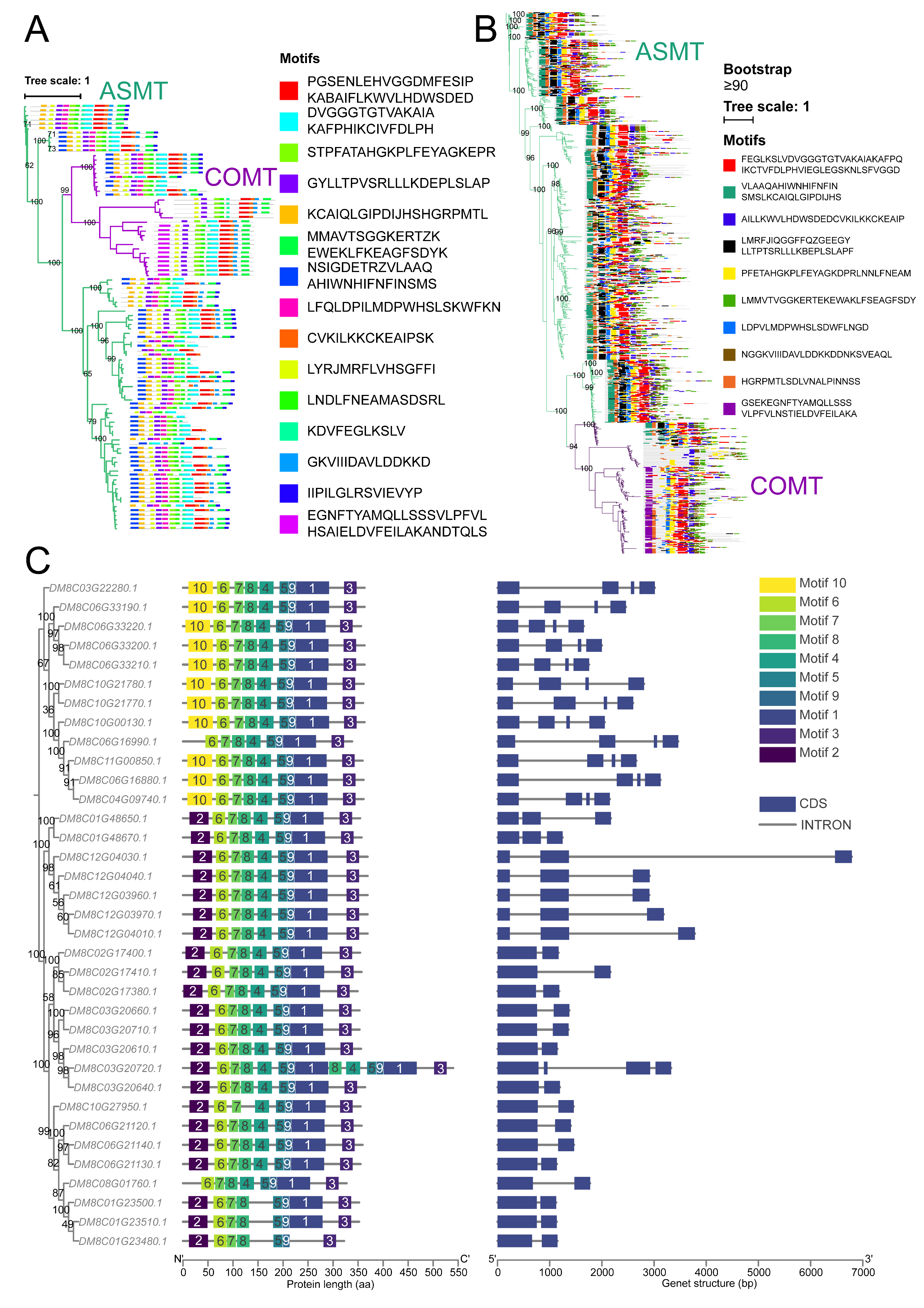


**Figure S6. Phylogenetic and motif analysis of the *StASMT/StCOMT* gene family in the pangenome of potato.**

**(A)** Phylogenetic and motif analysis of the *StASMT/StCOMT* gene family in diploid potato pangenome.

**(B)** Phylogenetic and motif analysis of the *StASMT/StCOMT* gene family in tetraploid potato pangenome.

**(C)** Motif and gene structure analysis of the *ASMT/COMT* gene family in *Solanum tuberosum*.


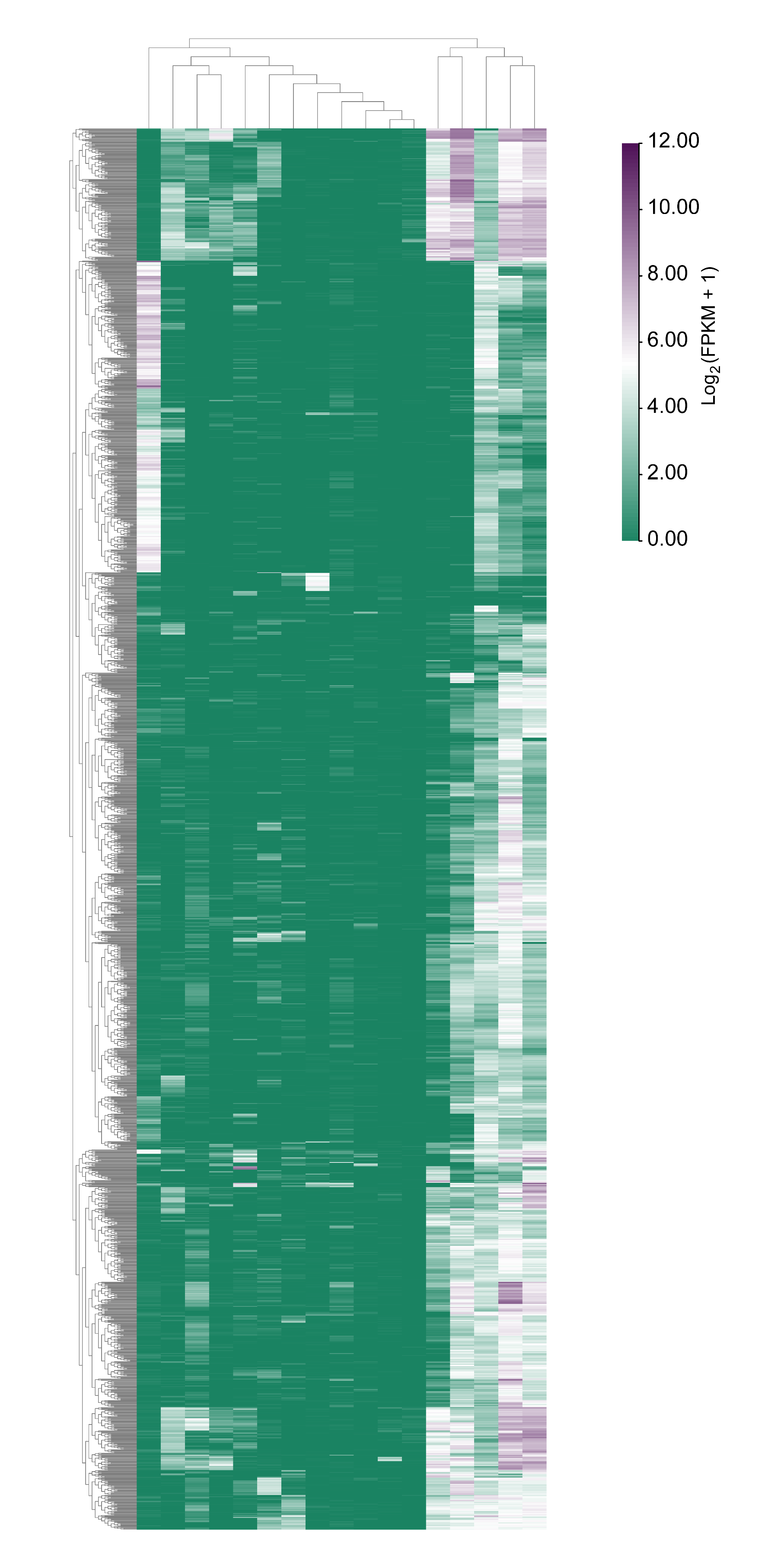


**Figure S7. Analysis of the expression patterns of the *ASMT/COMT* gene family.** *SlASMT/SlCOMT* expression patterns in tomato.

**Supplementary Tables**

**Table S1.** Large-scale Analysis Result of *ASMT/COMT* Gene Family Basic Sequence Information. The table contains the ids corresponding to the identified gene family members, the e-values corresponding to BLAST and Hmm search, and the protein sequences.

**Table S2.** Large-scale Analysis Result of *ASMT/COMT* Gene Family Groups Numbers. Different columns represent copies of different subgroups in different species.

**Table S3.** Mutation Prediction of Key Differentiation Sites in ASMT/COMT.

**Table S4.** Large-scale Analysis Result of Melatonin Receptor *CAND2* Gene Family Basic Sequence Information. The table contains the ids corresponding to the identified gene family members, the e-values corresponding to BLAST and Hmm search, and the protein sequences.

**Table S5.** Large-scale Analysis Result of Melatonin Receptor *CAND2* Copy Numbers

**Table S6.** *ASMT/COMT* Gene Family in *Arabidopsis thaliana* Pan-genome. This Table records the basic information of ASMT/COMT, which is close to the content of Table S1, but has the clustering results that emerged from pan-genome analysis.

**Table S7.** *ASMT/COMT* Homologous Gene Pairs in 104 Poaceae Species.

**Table S8.** *ASMT/COMT* Homologous Gene Pairs’ Ka, Ks and Ka/Ks in 104 Poaceae Species.

**Table S9.** *ASMT/COMT* Collinearity Analysis Result of Wheat Sub-chromosomes.

**Table S10.** *ASMT/COMT* Homologous Gene Pairs and Their Chromosomal Location in *Triticum aestivum* ‘Chinese Spring’.

**Table S11.** *ASMT/COMT* Gene Family in Wheat Pan-genome. Similar to the recording method of Table S6.

**Table S12.** *ASMT/COMT* Orthologous Gene Groups (OGGs) Genes’ Numbers and Types in Wheat Pan-genome.

**Table S13.** *ASMT/COMT* Gene Family in *Ae. tauschii* Pan-genome. Similar to the recording method of Table S6.

**Table S14.** *ASMT/COMT* Homologous Gene Pairs’ Ka, Ks and Ka/Ks in Wheat Pan-genome.

**Table S15.** *ASMT/COMT* Gene Family in Potato Pan-genome. Similar to the recording method of Table S6.

**Table S16.** *ASMT/COMT* Homologous Gene Pairs in 88 Solanaceae Species.

**Table S17.** *ASMT/COMT* Homologous Gene Pairs’ Ka, Ks and Ka/Ks in Potato.

**Table S18.** *ASMT/COMT* Heatmap Raw Data (FPKM) in Figure 6.

**Table S19.** *ASMT/COMT* Gene Family Renaming Table in Wheat in Figure S3
