# Supplementary figures and images for: Large-scale plant genomic identification and analysis uncover ASMT/COMT copy number variation driving melatonin dosage balance
Source: Hortic Res. 2025 Dec 18;13(3):uhaf348. doi: 10.1093/hr/uhaf348 (PMC13002329; doi:10.1093/hr/uhaf348)

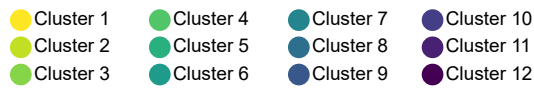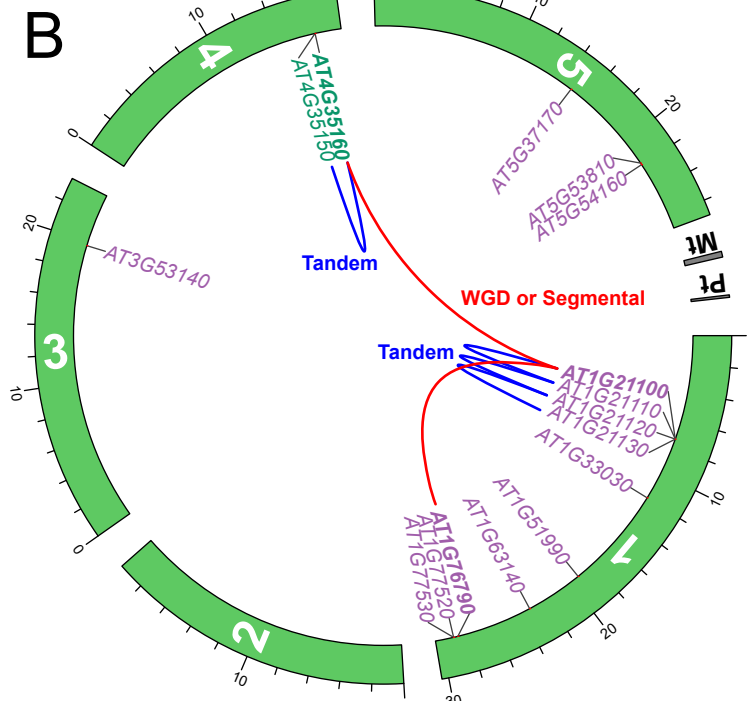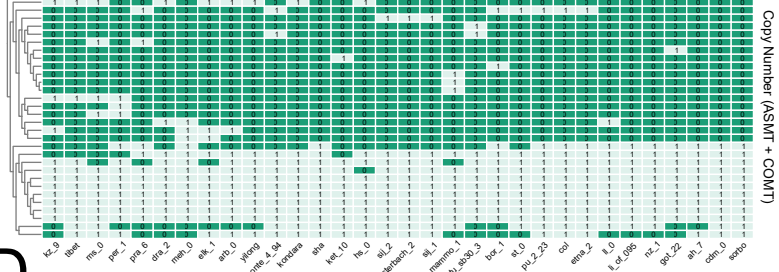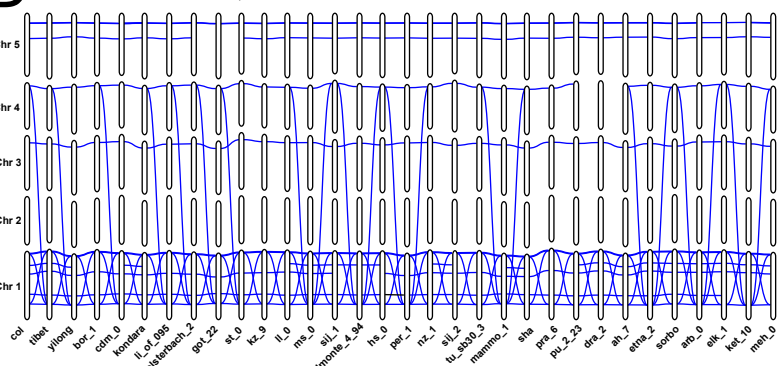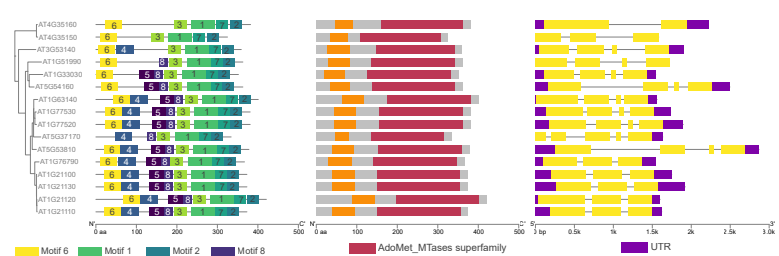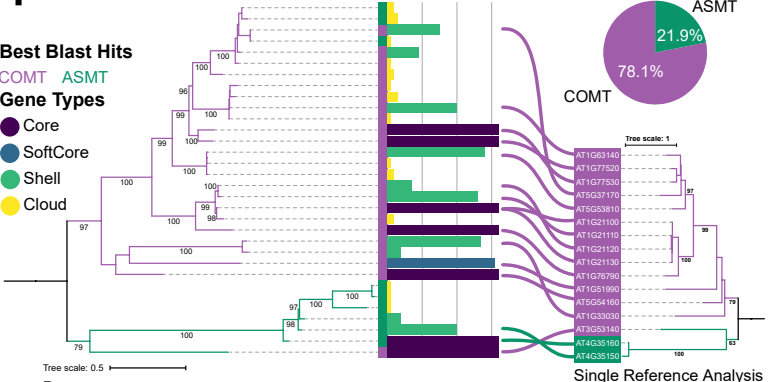

Supplement: Web_Material_uhaf348 [file web_material_uhaf348.zip › Figure_S1.pdf]

**A**

Cloud 2.8% Softcore 8.2%

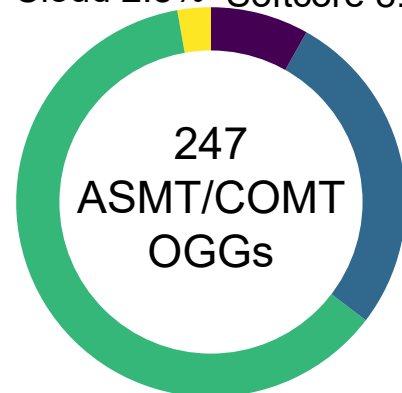

Core 61.8% Shell 27.2%

**B**

Copy Number (ASMT + COMT)

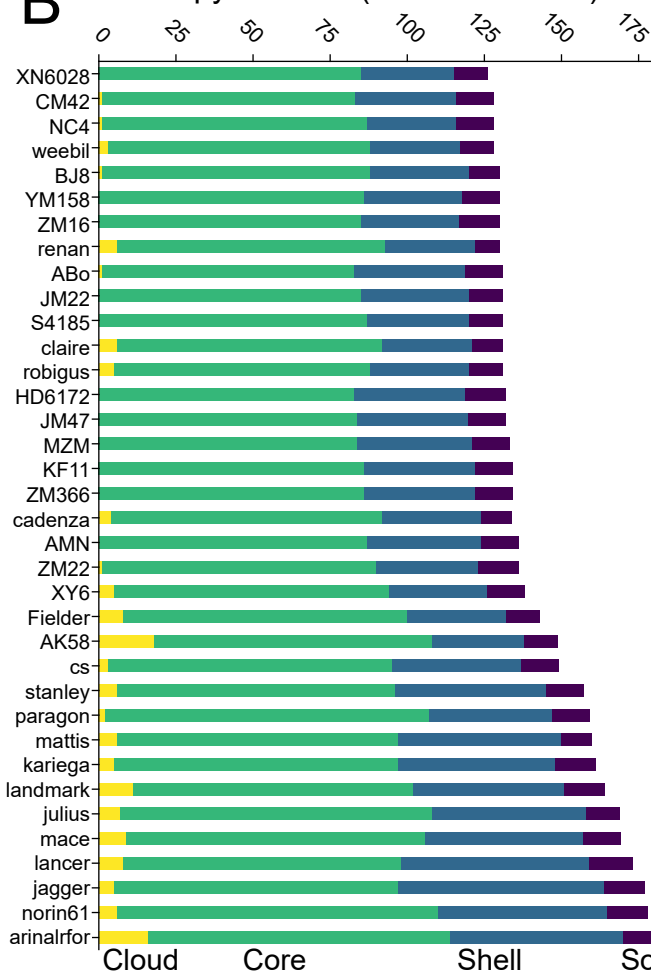**C**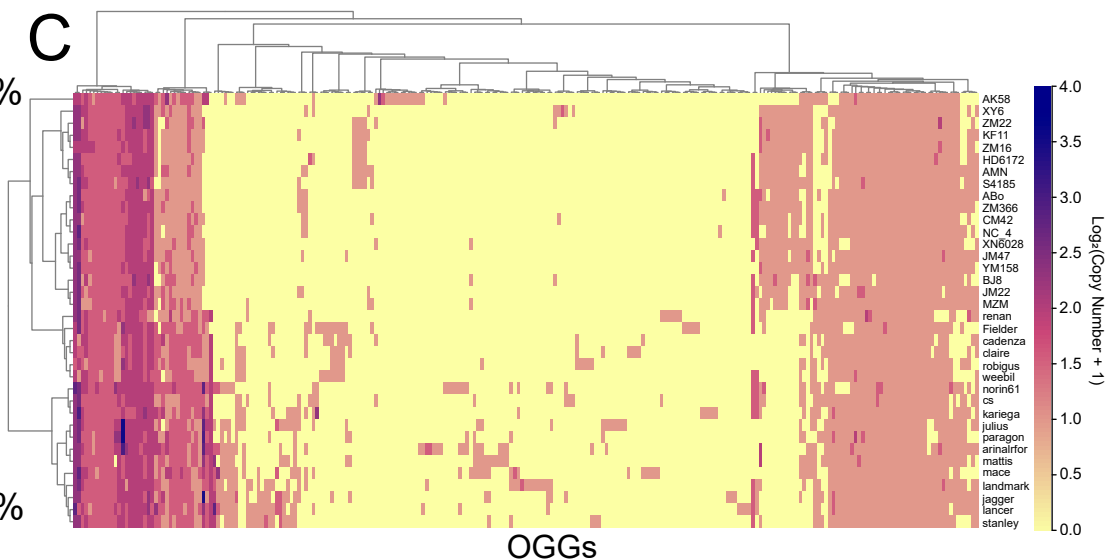**D**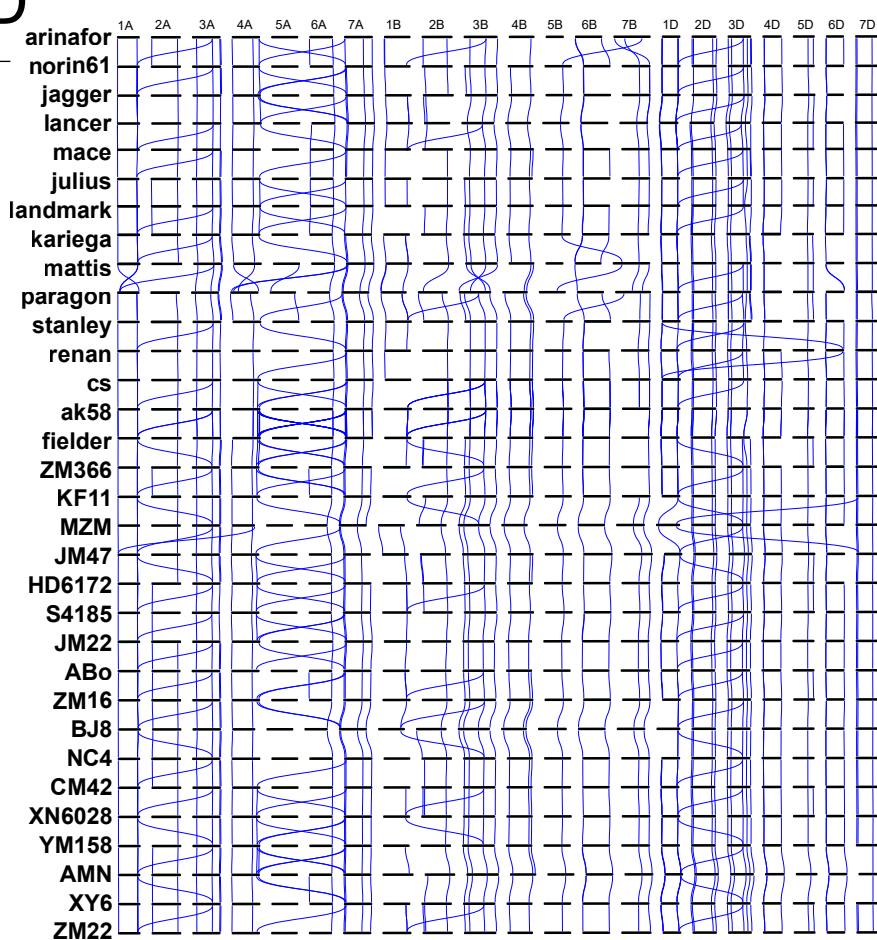

Supplement: Web_Material_uhaf348 [file web_material_uhaf348.zip › Figure_S2.pdf]

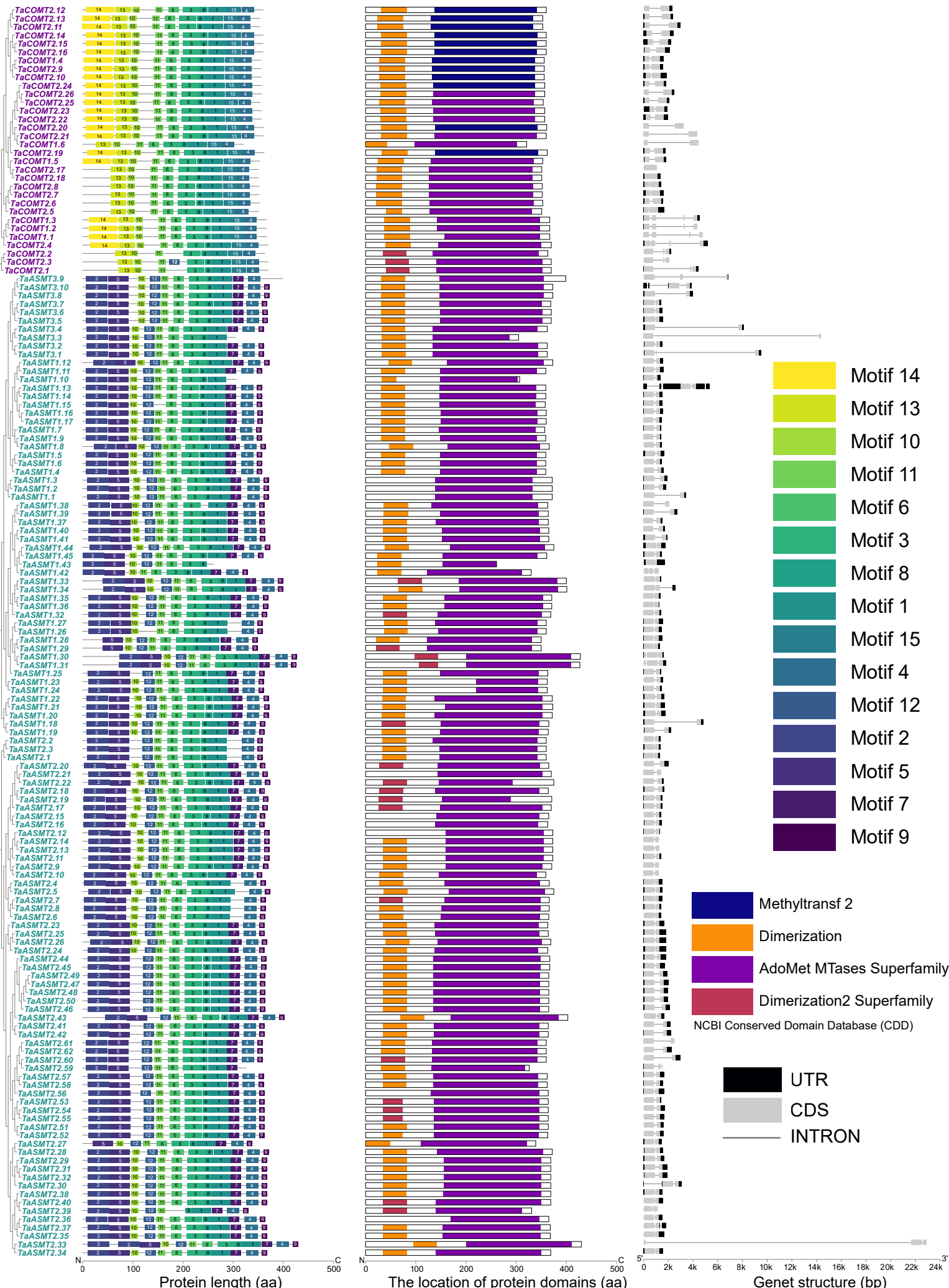

Supplement: Web_Material_uhaf348 [file web_material_uhaf348.zip › Figure_S3.pdf]

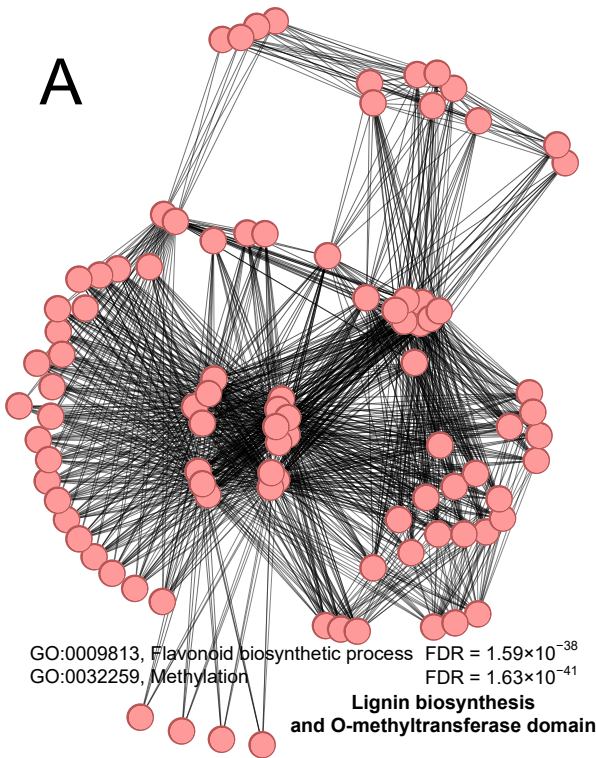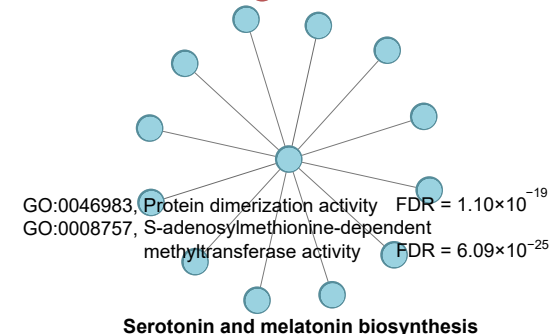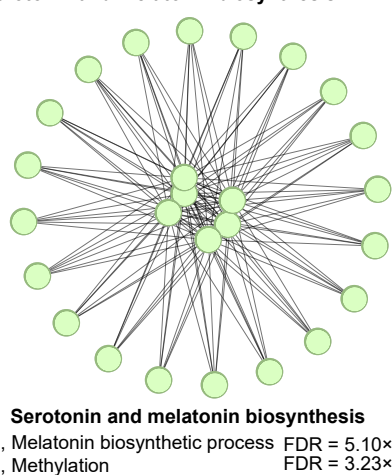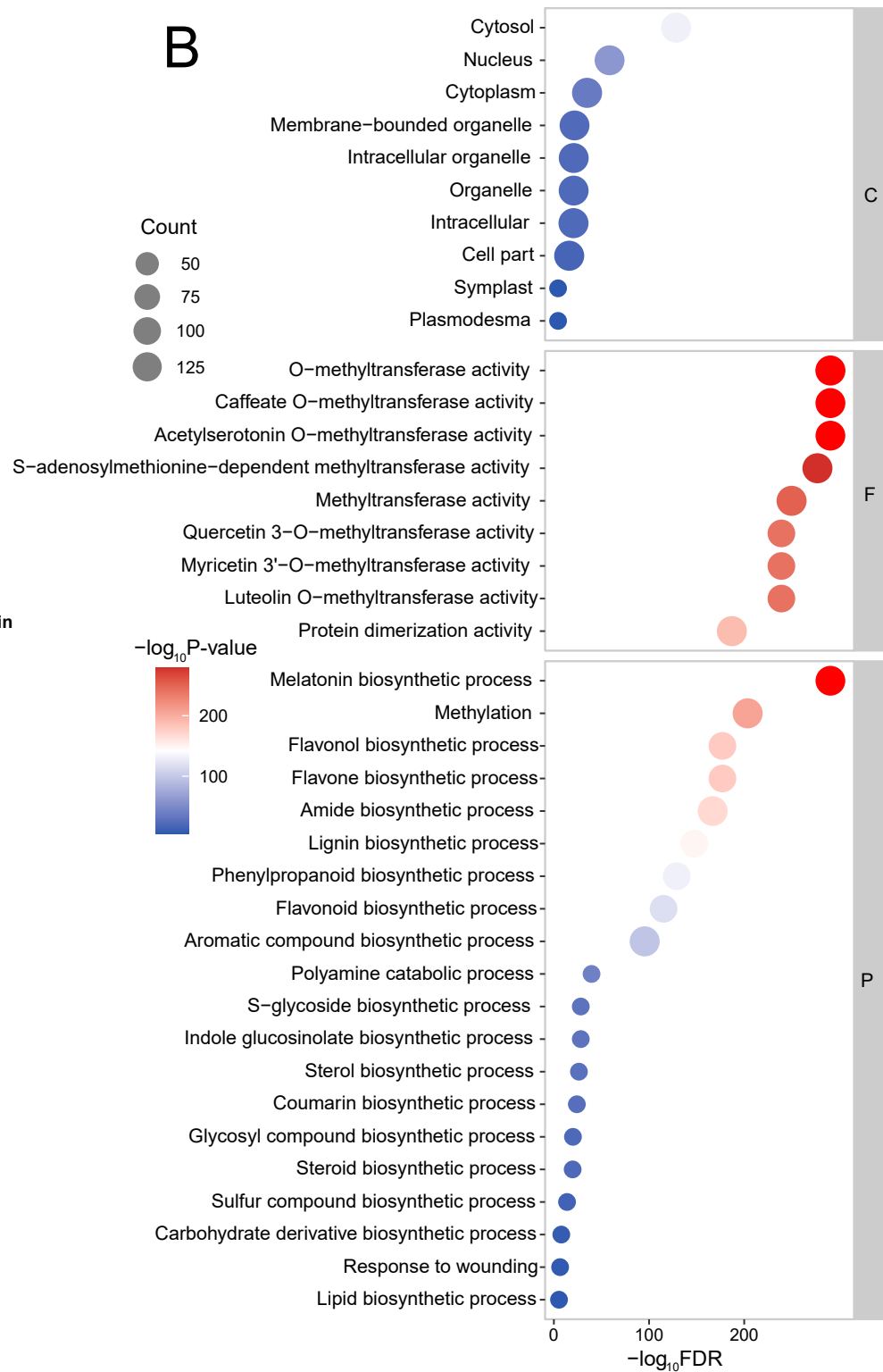

Supplement: Web_Material_uhaf348 [file web_material_uhaf348.zip › Figure_S5.pdf]

A

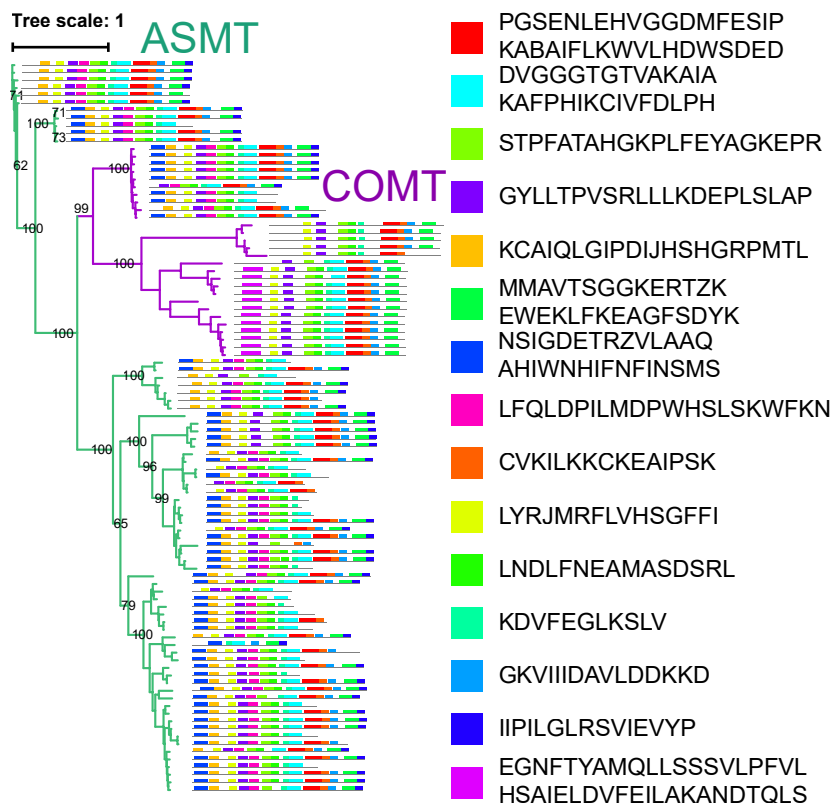

B

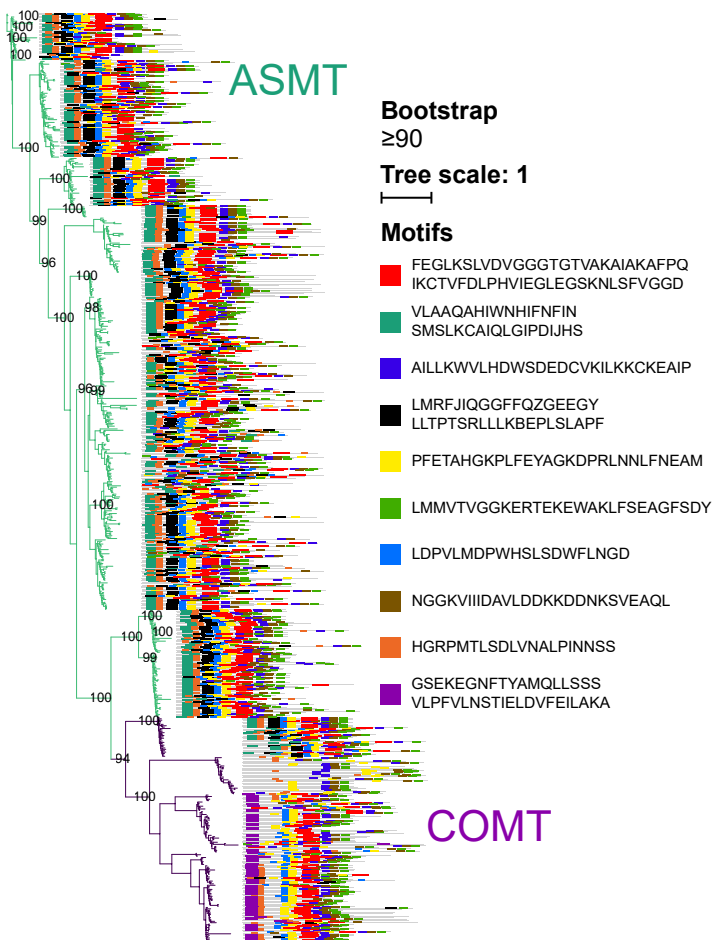

C

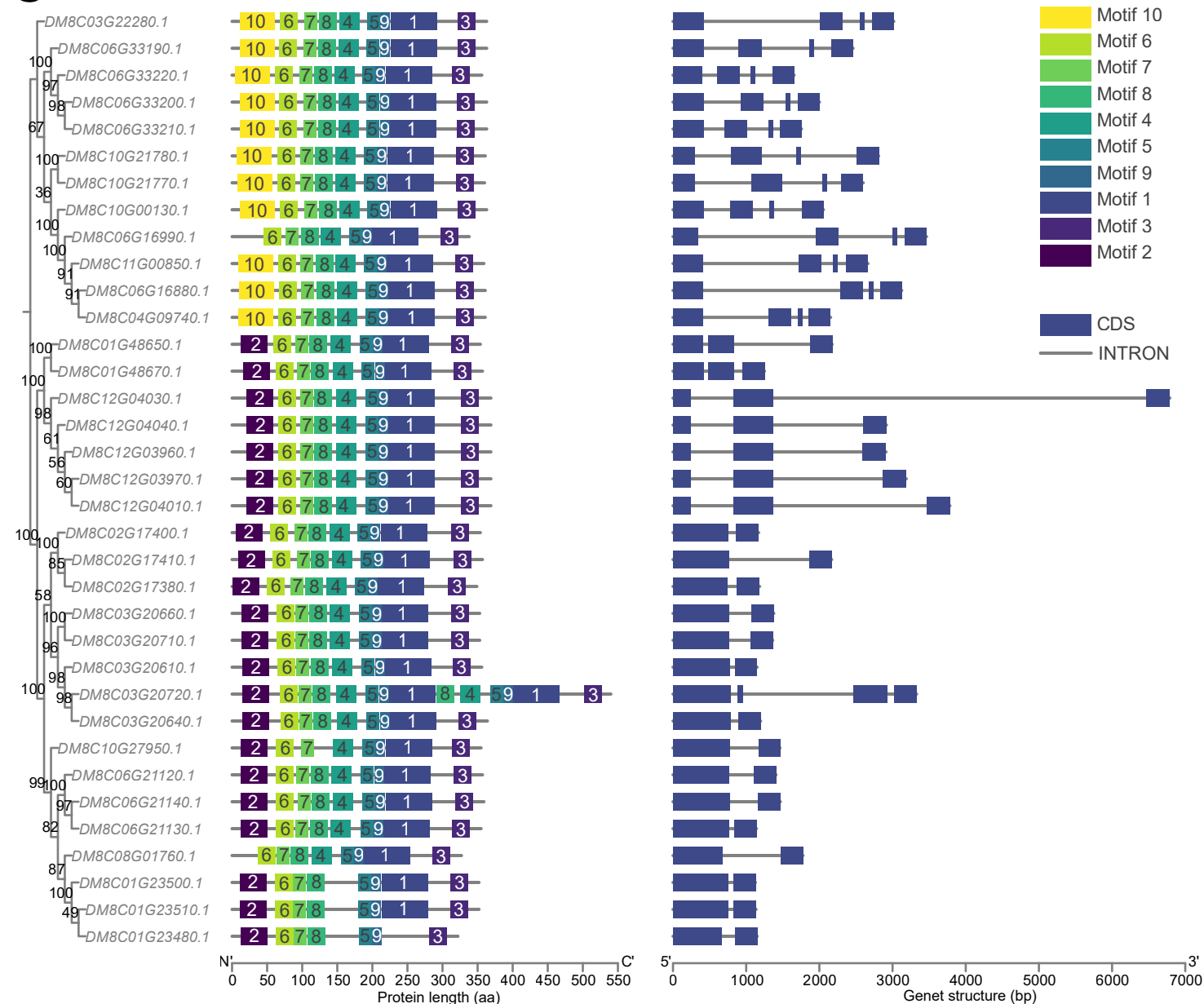

Supplement: Web_Material_uhaf348 [file web_material_uhaf348.zip › Figure_S6.pdf]

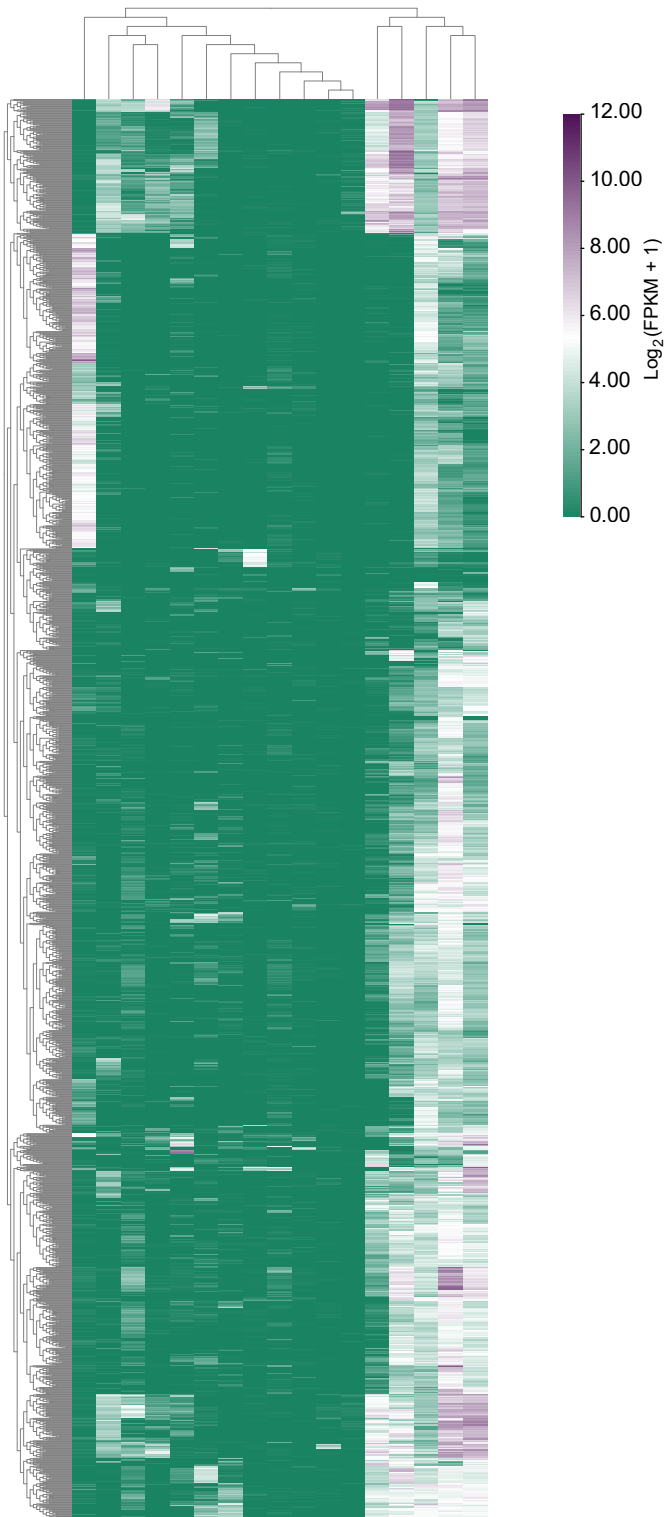

Supplement: Web_Material_uhaf348 [file web_material_uhaf348.zip › Figure_S7.pdf]
